# Expanding the Toolkit of Fluorescent Biosensors for Studying Mitogen Activated Protein Kinases in Plants

**DOI:** 10.3390/ijms21155350

**Published:** 2020-07-28

**Authors:** Kati Seitz, Patrick J. Krysan

**Affiliations:** 1 Laboratory of Genetics, University of Wisconsin-Madison, Madison, WI 53706, USA; kaseitz@wisc.edu; 2 Horticulture Department, University of Wisconsin-Madison, 1575 Linden Drive, Madison, WI 53706, USA

**Keywords:** plant MAP Kinase, MAPK docking domain, fluorescent biosensor, FRET biosensor, translocation biosensor

## Abstract

Mitogen-activated protein kinases (MAPKs) are key regulators of numerous biological processes in plants. To better understand the mechanisms by which these kinases function, high resolution measurement of MAPK activation kinetics in different biological contexts would be beneficial. One method to measure MAPK activation in plants is via fluorescence-based genetically-encoded biosensors, which can provide real-time readouts of the temporal and spatial dynamics of kinase activation in living tissue. Although fluorescent biosensors have been widely used to study MAPK dynamics in animal cells, there is currently only one MAPK biosensor that has been described for use in plants. To facilitate creation of additional plant-specific MAPK fluorescent biosensors, we report the development of two new tools: an in vitro assay for efficiently characterizing MAPK docking domains and a translocation-based kinase biosensor for use in plants. The implementation of these two methods has allowed us to expand the available pool of plant MAPK biosensors, while also providing a means to generate more specific and selective MAPK biosensors in the future. Biosensors developed using these methods have the potential to enhance our understanding of the roles MAPKs play in diverse plant signaling networks affecting growth, development, and stress response.

## 1. Introduction

Conserved across all eukaryotes [1], mitogen-activated protein kinases (MAPKs) have been shown to play critical roles in a wide array of biological processes. Within the plant model system *Arabidopsis thaliana*, MAPKs have been implicated in many aspects of plant life, including cell division [2,3], abiotic stress responses [4,5,6,7,8,9], biotic stress responses [10,11,12,13,14,15,16,17], developmental patterning [18,19,20], stomatal closure [21,22], and many other pathways [23,24,25]. Although biochemical methods to measure MAPK signaling have provided valuable insight into MAPK function within these pathways, these methods generally report the average MAPK activity within a population of cells and cannot provide cell-level resolution of the spatiotemporal dynamics these kinases display in different biological contexts.

One approach that can allow one to measure MAPK activation with greater resolution involves the use of fluorescence-based genetically-encoded biosensors [26,27]. Fluorescent biosensors provide an easily quantifiable, real-time readout of a targeted parameter in living cells, elucidating the roles of different biomolecules in the complex dynamics that underlie cellular networks [28]. A variety of fluorescent biosensors have been developed to shed light on the mechanisms by which MAPKs function and are regulated in animal cells [27]; the most common of these biosensors are Förster resonance energy transfer (FRET) biosensors [26,29,30,31,32,33,34,35,36].

A FRET biosensor that measures MAPK activity is a single polypeptide that contains: two fluorophore domains that are able to engage in FRET, a phosphoamino acid binding domain, a linker region, and a substrate domain. The substrate domain itself contains two important functional elements: a MAPK docking domain and a phosphoacceptor site. In the absence of active MAPK, the FRET biosensor is expected to exist in a non-phosphorylated state. In this condition, the phosphoamino acid binding domain has low affinity for interaction with the phosphoacceptor domain. If active MAPK is present, however, then the FRET biosensor is expected to become phosphorylated at the phosphoacceptor site. A conformational shift driven by binding of the phosphoamino acid binding domain to the phosphorylated substrate domain occurs, resulting in an increase in FRET efficiency. This change in FRET can be measured real-time in living tissue using confocal microscopy, with the change in FRET serving as a readout of MAPK activation in vivo. Because of the high resolution with which confocal microscopes can image living tissue, changes in MAPK activity can be observed with single-cell resolution. Changes in FRET produced by these biosensors in response to phosphorylation can also be measured in vitro using an instrument such as a spectrofluorometer. By implementing these MAPK FRET biosensors within animal systems, researchers have mapped aspects of MAPK activity such as the duration or amplitude of activation [37], heterogeneity of response at the cell population level [38,39] or the subcellular level [40], and fluctuations in activity such as oscillations [35,41], greatly enriching comprehension of MAPK spatiotemporal dynamics within these cells [42,43].

The first plant MAPK fluorescent biosensor, the FRET-based sensor of MAPK in *Arabidopsis* (SOMA) was recently described [44]. SOMA has been shown to report the activities of the *Arabidopsis* MAPKs MPK3 and MPK6, and it is also likely to report the activities of additional MAPK isoforms in *Arabidopsis*, such as MPK4 [44]. As the *Arabidopsis* genome encodes 20 MAPK isoforms, there remains a need for additional plant MAPK biosensors that report the activities of different MAPK isoforms. It would also be of great value to have biosensors with the specificity to report the activity of a single MAPK isoform. For these reasons, the plant MAPK research community would benefit from the ability to efficiently develop new plant MAPK biosensors. However, development of a new FRET biosensor tends to require a considerable amount of time, as no universal design rules exist to guide the optimization process [45,46,47,48,49,50]. For this reason, we felt that it would be valuable to establish a set of tools that would streamline the process of developing new plant MAPK biosensors.

For a MAPK FRET biosensor to be useful, all of the functional domains that comprise that biosensor must work together to produce a protein that undergoes an appropriate conformational shift in response to phosphorylation. One of the most important determinants of that conformational shift is the interaction between the phosphoamino acid binding domain and the phosphorylated phosphoacceptor site. When designing FRET biosensors for a specific MAPK isoform, the standard approach involves testing a number of candidate substrate domains that contain both a phosphoacceptor site and a docking domain that facilitates association of the substrate with the target MAPK [26,47,48,49]. By doing so, one hopes to find a substrate domain that can not only be efficiently phosphorylated by the MAPK, but that also has the ability to efficiently interact with the phosphoamino acid binding domain of the FRET biosensor. Unfortunately, this approach requires screening candidate sequences for their ability to simultaneously meet two functional criteria: (1) the ability to be efficiently phosphorylated, and (2) the ability to effectively interact with the phosphoamino acid binding domain. Reducing the complexity of this process so that new sequences are screened using only a single functional criterion would make it possible to more efficiently develop new plant MAPK biosensors. The approach we took to accomplish this goal was to re-purpose a previously optimized mammalian MAPK FRET biosensor to serve as a plant MAPK docking domain trap.

The plant MAPK docking domain trap that we developed allowed us to screen for plant amino acid sequences that facilitate efficient phosphorylation of a mammalian MAPK phosphoacceptor site. Because the two fluorophores, the linker, the phosphoacceptor site, and the phosphoamino acid binding domain of this plant MAPK docking domain trap have been pre-optimized to produce a strong FRET change in response to phosphorylation, the only functional contribution required from the plant sequences was the ability to facilitate the binding of the MAPK to the biosensor. In the present study we report how this plant MAPK docking domain trap can be deployed using an in vitro assay to efficiently screen for new plant MAPK docking domains.

In order to diversify the variety of tools available to plant scientists for studying MAPK activity in vivo, we sought to determine if an alternative to FRET-based biosensors could be established for use in plants. To accomplish this, we turned to a biosensor variant used to study kinase activation in animal cells called a translocation biosensor [51]. This type of biosensor possesses both a nuclear localization signal (NLS) module and a nuclear exclusion signal (NES) module, with several phosphorylation sites scattered throughout [51]. The phosphorylation status of these sites impacts the activities of these localization signals, directing the biosensor to either localize or be excluded from the nuclei of cells in a phosphorylation-dependent manner. By attaching a docking domain targeted by a kinase of interest to this type of biosensor, the nucleocytoplasmic shuttling of the sensor can serve as a visual indicator of kinase activity [51]. Translocation biosensors offer several potential benefits in comparison to FRET-based biosensors and have proven to be powerful tools for documenting kinase spatiotemporal dynamics in different animal models [51,52,53,54,55].

In the present study we demonstrate that the translocation biosensor strategy described above is applicable to plants by showing that the nuclear localization activity of the bNLS/NES domain can be modulated by phosphomimetic mutations. We also demonstrate that inclusion of a plant MAPK docking domain in the translocation biosensor construct produces a protein that shifts from nuclear to cytoplasmic localization in response to treatments known to elicit MAPK activation in plants. Taken together, these results suggest that the kinase translocation biosensor approach developed for studying animal cell MAPK activation can also be effectively deployed for studying plant MAPKs.

The design of these translocation biosensors is such that the only functional element that needs to be added to the biosensor backbone to produce the functional biosensor is a docking domain specific for the kinase of interest. When paired with the docking domain trap described earlier, this combination of tools produces a compact toolkit for efficiently generating new plant MAPK biosensors. The docking domain trap can be used to rapidly screen in vitro for amino acid sequences that specifically interact with chosen plant MAPK isoforms. Once those docking domains have been characterized in vitro, they can be deployed in vivo either in the context of the FRET biosensor backbone or transferred to the translocation biosensor backbone. Application of these tools should allow for enhanced exploration of the role of MAPK activation kinetics in many aspects of plant biology.

## 2. Results

### 2.1. Developing a FRET-Based Plant MAPK Docking Domain Trap

**Figure 1:**
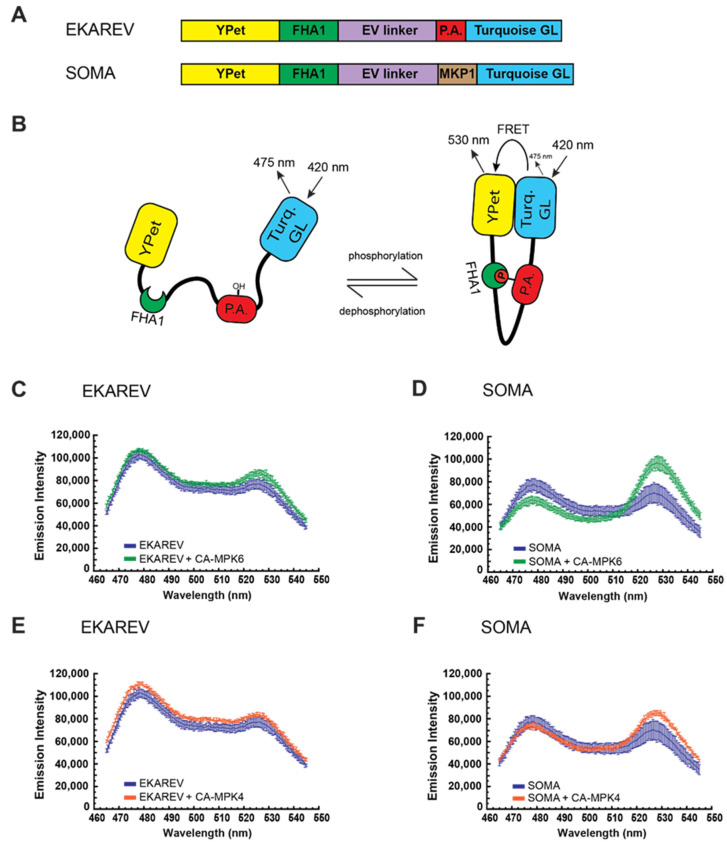
Development of the plant mitogen-activated protein kinase (MAPK) docking domain trap. (**A**) Domain structure of EKAREV and sensor of MAPK in *Arabidopsis* (SOMA). YPet is a yellow fluorescent protein, FHA is the FHA1 phosphopeptide binding domain of yeast RAD53, EV linker is a flexible linker domain, P.A. is the phosphoacceptor region of the mammalian CdC25C protein, MKP1 is an 80 amino acid region of the *Arabidopsis* MKP1 protein, and Turquoise GL is a blue fluorescent protein. Maps are not to scale. (**B**) Cartoon schematic of the EKAREV biosensor. Phosphorylation of EKAREV within the phosphoacceptor domain is expected to produce a conformation shift that increases Förster resonance energy transfer (FRET) efficiency between the Turquoise GL and YPet fluorophores due to the enhanced affinity of the FHA1 domain for the phosphorylated form of the phosphoacceptor domain. Removal of the phosphate is expected to cause EKAREV to revert to a conformation with lower FRET efficiency. (**C**–**F**) Data from in vitro FRET assays using EKAREV (**C**,**E**) or SOMA (**D**,**F**) in the presence or absence of constitutively active MPK6 (CA-MPK6) (**C**,**D**) or MPK4 (CA-MPK4) (**E**,**F**). The emission spectra of the biosensors in response to excitation with 420 nm light is shown. Three technical replications were performed for each experiment, and the values plotted are the means for those three trials with the error bars indicating standard deviation. Absence of a CA-MAPK is represented in blue. Presence of CA-MPK6 is represented in green. Presence of CA-MPK4 is represented in orange. Emission intensity units are arbitrary “counts per second”.

In order to establish a pipeline for efficiently developing new plant MAPK fluorescent biosensors, we first set out to establish an in vitro assay for efficiently identifying new plant MAPK docking domains. To do so, the approach that we took was to develop a vector that would serve as a plant MAPK docking domain trap. We produced this trap by re-purposing an established MAPK FRET biosensor called EKAREV [26,48] that had been proven effective for reporting MAPK activity in animal cells. The important functional domains of EKAREV are the YPet yellow fluorescent protein domain [56], the mTurquoise-GL cyan fluorescent protein domain [57], the EV linker [48], the FHA1 phosphoamino acid binding domain [58,59], a 14 amino acid phosphoacceptor domain from the mammalian Cdc25C protein [60], and a docking domain recognized by the human extracellular signal-regulated kinases (ERKs) [61] (Figure 1A), which are a type of MAPK. A cartoon schematic of EKAREV in action is shown in Figure 1B. As the EKAREV biosensor does not contain a plant-specific MAPK docking domain, we postulated that it may not be efficiently phosphorylated by plant MAPKs. If so, this FRET biosensor had the potential to serve as a plant MAPK docking domain trap. To test this hypothesis, we used an in vitro assay that we previously developed for evaluating candidate plant MAPK FRET biosensors [44].

For this in vitro assay, biosensor protein and constitutively active versions of the *Arabidopsis* MAPKs MPK4 and MPK6 (CA-MPK4 and CA-MPK6) are expressed as recombinant proteins using *Escherichia coli* [44,62]. Recombinant biosensor protein is then incubated together with constitutively active kinase and adenosine triphosphate (ATP), and the reactions are subsequently analyzed using a scanning spectrofluorometer to measure the fluorescent emission generated by illumination of the samples at a wavelength that excites the mTurquoise-GL FRET donor. When EKAREV was incubated in the presence of CA-MPK6, we observed a ca. 9% increase in FRET efficiency when compared to the control reaction that did not contain kinase (Figure 1C, Table 1). This FRET gain was substantially lower than the ca. 68% increase in FRET that CA-MPK6 induced in our previously described *Arabidopsis* MAPK biosensor SOMA (Figure 1D, Table 1). These results suggested that CA-MPK6 may have only a limited capacity to phosphorylate EKAREV. Next, we repeated the above experiment using CA-MPK4 and found that this kinase caused no FRET gain for EKAREV (Figure 1E). In the control reaction, we observed that CA-MPK4 induced a ca. 27% increase in the FRET efficiency of SOMA (Figure 1F).

### 2.2. Testing the Plant MAPK Docking Domain Trap

We hypothesized that the low FRET gain induced in EKAREV by CA-MPK6 and the lack of a FRET gain caused by CA-MPK4 could be explained by at least two different mechanisms. First, the mammalian phosphoacceptor domain present in EKAREV may not be able to be efficiently targeted by CA-MPK4/6. Alternatively, the mammalian MAPK docking domain present in EKAREV may not efficiently recruit CA-MPK4/6 to bind to the biosensor. To explore these two possibilities, we inserted the DNA sequence that encoded a known *Arabidopsis* MAPK docking domain into the EKAREV vector.

**Figure 2:**
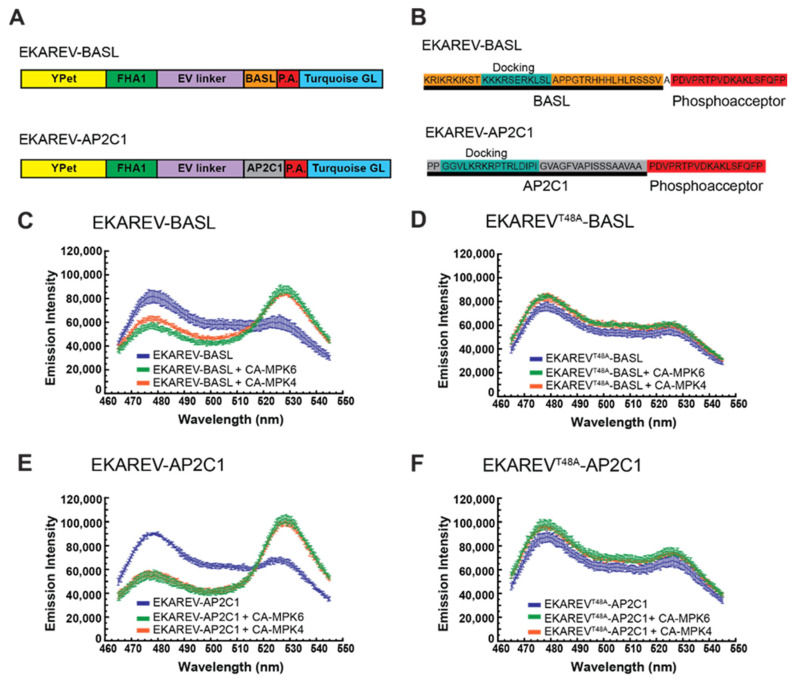
Inserting plant MAPK docking domains into EKAREV allows for large MAPK-induced increases in FRET. (**A**) Domain structure of EKAREV-BASL and EKAREV-AP2C1. YPet is a yellow fluorescent protein, FHA is the FHA1 phosphopeptide binding domain of yeast RAD53, EV linker is a flexible linker domain, P.A. is the phosphoacceptor region of the mammalian CdC25C protein, BASL is a 39 aa region of the plant BASL protein that contains a plant MAPK docking domain. AP2C1 is a 35 a region of the AP2C1 protein that contains a plant MAPK docking domain. Maps are not to scale. (**B**) Protein sequence for the docking domain and phosphoacceptor regions of each biosensor. Sequence from the BASL protein is highlighted in orange, sequence from the AP2C1 protein is highlighted in grey, and the EKAREV phosphoacceptor domain is highlighted in red. The docking domains are highlighted in blue-green. (**C**–**F**), Data from in vitro FRET assays using EKAREV-BASL (**C**), EKAREV^T48A^-BASL (**D**), EKAREV-AP2C1 (**E**), or EKAREV^T48A^-AP2C1 (**F**) in the presence or absence of constitutively active MPK6 (CA-MPK6) or MPK4 (CA-MPK4). The emission spectra in response to excitation at 435 nm is shown. The values plotted are the means of three technical replicates of each experiment, with the error bars indicating standard deviation. Emission intensity units are arbitrary “counts per second”.

For this experiment we used a region of the coding sequence from the *Arabidopsis* breaking of asymmetry in the stomatal lineage (*BASL*) gene [63]. The BASL protein has been previously characterized as a MPK3/6 substrate, and several plant MAPK docking domains have been mapped on its structure [64,65]. We used a section of the *BASL* gene encoding 39 amino acids that contained the D1 docking domain and inserted it into the EKAREV plasmid, resulting in EKAREV-BASL (Figure 2A,B). Incubating EKAREV-BASL with CA-MPK6 resulted in a ca. 115% gain in FRET, whereas CA-MPK4 induced a ca. 85% gain (Figure 2C, Table 1). Our observation that EKAREV-BASL exhibited substantially higher MAPK-induced FRET increases than EKAREV is consistent with the hypothesis that adding a suitable *Arabidopsis* MAPK docking domain to EKAREV is sufficient to allow the biosensor to be efficiently phosphorylated by plant MAPKs.

To determine if the mammalian phosphoacceptor domain present in EKAREV was being targeted by CA-MPK4/6, we mutagenized the threonine residue in EKAREV-BASL thought to behave as the phosphorylation site into an alanine [26,48,60]. The resulting mutant version of the biosensor, EKAREV^T48A^-BASL, showed no increase in FRET efficiency when incubated with either CA-MPK4 or CA-MPK6 (Figure 2D, Table 1), similar to the results observed when the phosphorylation site of SOMA was mutagenized to an alanine (Figure S1A,B, Table 1) [44,66,67]. These results demonstrate that the MAPK-induced FRET gain observed with EKAREV-BASL is dependent on the threonine present within the EKAREV phosphoacceptor domain and suggest that the increase in FRET efficiency is likely caused by phosphorylation of that threonine by CA-MPK4/6.

The results described above suggested that EKAREV has the potential to serve as a plant MAPK docking domain trap. To further test this hypothesis, we cloned the DNA sequence encoding another previously characterized plant MAPK docking domain into the EKAREV vector. We used a region of the *Arabidopsis* PP2C-type phosphatase AP2C1, which is a characterized MPK6 substrate that contains verified MAPK docking domains [68,69]. When a region of the *AP2C1* gene encoding one such docking domain was added to the EKAREV backbone (Figure 2A,B), the resulting biosensor, EKAREV-AP2C1, exhibited an increase in FRET efficiency of ca. 150% in response to CA-MPK6 and an increase of ca. 140% in response to CA-MPK4 (Figure 2E, Table 1). Mutagenizing the predicted phosphorylation site within the EKAREV phosphoacceptor domain eliminated this effect (Figure 2F, Table 1). Therefore, incorporating a different plant MAPK docking domain into EKAREV also produced a biosensor that was able to report MAPK-induced FRET increases when treated with plant MAPKs. Taken together with the results obtained with EKAREV-BASL, these results indicate that EKAREV is able to function as a plant MAPK docking domain trap.

By establishing EKAREV as a plant MAPK docking domain trap, we have developed a tool for functionally assessing candidate *Arabidopsis* MAPK docking domains. It should also be noted that the EKAREV-docking domain constructs that we produced are also functional FRET-based biosensors which can be directly moved into plants for evaluation of their potential to serve as in vivo biosensors for MAPK activity in living plant tissue. In addition, the plant MAPK docking domains identified using this in vitro assay likely possess some degree of modularity such that they could potentially be incorporated into other types of fluorescent biosensors, as described below.

### 2.3. Developing a Translocation-Based Biosensor for Use in Arabidopsis

One alternative to FRET-based biosensors for studying MAPK activity in vivo are translocation biosensors, or kinase translocation reporters (KTRs) [51]. KTRs are distinct from FRET-based kinase biosensors in that the subcellular localization of the fluorescent signal is the main readout of kinase activity. KTRs possess a bipartite nuclear localization signal (bNLS)/nuclear exclusion signal (NES) module, with several phospho-sites scattered through the bNLS sequence. In an unphosphorylated state, the activity of the bNLS is dominant, resulting in the biosensor localizing to the nucleus of the cell. However, upon phosphorylation, the activity of the bNLS is repressed and the activity of the NES is enhanced, causing the biosensor to be excluded from the nucleus and accumulate in the cytoplasm. If a docking domain is added to the KTR that allows the biosensor to be phosphorylated by a kinase of interest, the subcellular localization of the resulting biosensor reflects the activation state of the chosen kinase.

Although relatively recently established, KTRs offer several potential advantages in comparison to FRET biosensors, such as a wider dynamic range and seemingly more accurate reporting of the downregulation of kinase activity [51,53]. Although KTRs have been successfully implemented in mammalian cells, *Caenorhabditis elegans,* and zebrafish [51,52,53,54,55], their use has not yet been reported in plants. In order to expand the set of fluorescent reporters available for studying MAPK activation in plants, we were therefore interested in determining if the KTR principle could be applied to *Arabidopsis*.

To create a translocation biosensor for reporting MAPK activity in plants, we began with the nuclear kinase translocation reporter (nKTR) developed for imaging MAPK activity in *C. elegans* [53]. nKTR contains a 44 amino acid docking domain derived from the mammalian Ets-like protein 1 (Elk1) [70,71], a bNLS containing two phospho-sites (S55 and S62), a NES, a FQFP motif [61], and the green fluorophore mClover [72]. The subcellular localization of the nKTR protein when expressed in *C. elegans* is driven by the equilibrium between the competing activities of the bNLS and NES domains, with the phosphorylation status of the bNLS capable of shifting the balance between nuclear localization and nuclear exclusion. Unphosphorylated nKTR was shown to localize to the nuclei of *C. elegans* germline cells, whereas phosphorylated nKTR was excluded from the nucleus [53].

**Figure 3:**
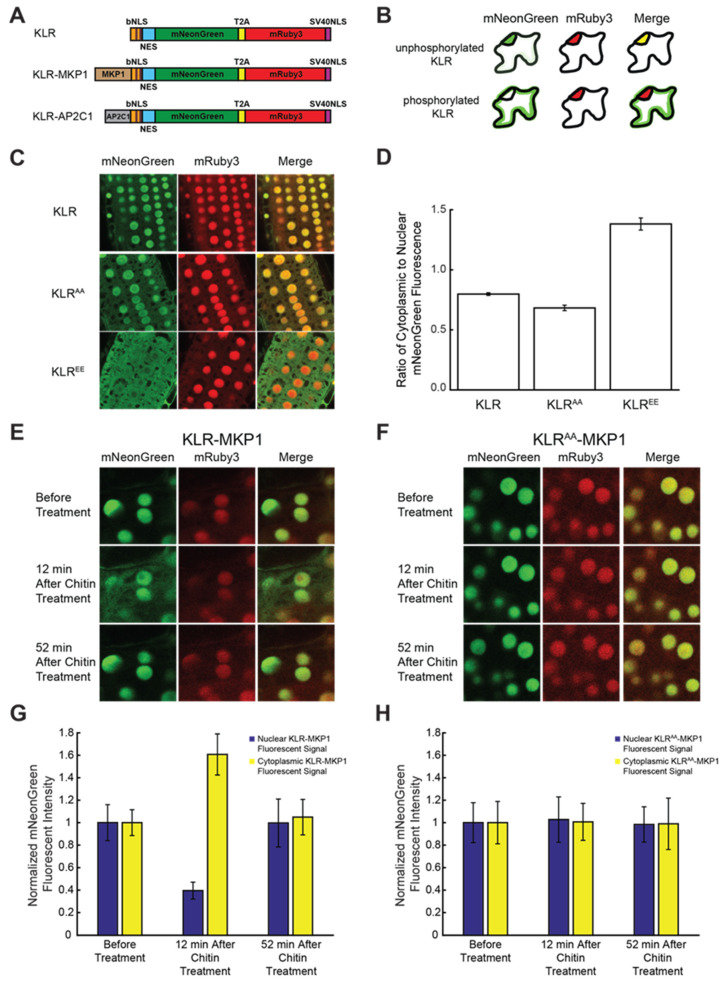
Functional characterization of the kinase localization reporter (KLR) kinase translocation reporter in *Arabidopsis* root cells. (**A**) Domain structure of KLR, KLR-MKP1, and KLR-AP2C1. bNLS is a bipartite modified nuclear localization signal derived from mouse C-Jun protein. The two phospho-acceptor sites (S63 and S73) are indicated by brown lines. NES is a consensus nuclear exclusion signal. mNeonGreen is a green fluorescent protein. T2A refers to the “self-cleaving” *Thosea asigna* virus 2A peptide. mRuby3 is a red fluorescent protein. SV40NLS is an NLS derived from simian virus 40 (SV40). Maps are not to scale (**B**) Cartoon of predicted KLR and mRuby3 localization. Unphosphorylated KLR is expected to localize to the nucleus, whereas phosphorylated KLR should be excluded from the nucleus. The nuclear marker mRuby3 is expected to constitutively localize to the nucleus. (**C**) mNeonGreen and mRuby3 emission in root tips of *Arabidopsis* seedlings expressing either KLR, KLR^AA^, or KLR^EE^ were imaged using confocal microscopy at 40x optical magnification and emission intensity was plotted in (**D**). Values plotted in (**D**) are the average cytoplasmic mNeonGreen emission divided by the average mNeonGreen nuclear emission. (**E**,**F**) Root tips of *Arabidopsis* seedlings expressing ether KLR-MKP1 (**E**) or KLR^AA^-MKP1 (**F**) were treated with 40 mg/mL chitin, and the mNeonGreen and mRuby3 emission channels were imaged using confocal microscopy at 40× optical magnification at the indicated timepoints and emission intensity was plotted in (**G**,**H**). The emission intensity values in (**G**,**H**) were normalized to the corresponding “before treatment” values. The quantitative data shown in (**D**,**G**,**H**) were obtained by measuring emission intensity in 8 nuclear and 8 cytoplasmic regions of interest in each of 12 cells for each sample type. Error bars indicate standard deviation.

In order to create a plant-based KTR, we synthesized a vector based on nKTR, with the following modifications: (1) the region encoding the Elk1 docking domain was removed, (2) the coding sequence for the mClover fluorophore was replaced with coding sequence for mNeonGreen [73], and (3) the entire coding sequence of the construct was codon-optimized for expression in plants. We assigned the name kinase localization reporter (KLR) to this plant-focused reporter, to distinguish it from the KTRs used in animal cells. This KLR construct does not contain any MAPK docking domains (Figure 3A). Based on the behavior of nKTR, unphosphorylated KLR was expected to localize to the nucleus. Upon phosphorylation, KLR would be expected to shift its localization to the cytoplasm, whereas dephosphorylation should cause it to return to the nucleus (Figure 3B). Quantifying the intensity of mNeonGreen fluorescence within the nucleus versus cytoplasm should thereby serve as a measure of kinase activity in the *Arabidopsis* cells in which the KLR biosensor is expressed.

The design of the nKTR biosensor construct used in *C. elegans* also contained the coding sequence for the spectrally-distinct mCherry fluorophore linked to the histone 2B (H2B) sequence. The mCherry-H2B portion of the nKTR construct provided an internal control for quantifying nuclear fluorescence [53]. The protein coding sequences for nKTR and mCherry-H2B were joined by a self-cleaving peptide from *Thosea asigna* virus 2A (T2A) [74,75], an element which induces ribosomal pausing during translation. Inclusion of this T2A peptide causes the single mRNA transcript generated by the nKTR vector to produce nKTR and mCherry-H2B as separate polypeptides. This arrangement allows the two proteins to be produced in equimolar quantities, while permitting each to localize independently within the cell. When nKTR was expressed in *C. elegans*, mCherry-H2B was found to be constitutively present in the nucleus, whereas the localization of nKTR was determined by the phosphorylation status of the reporter [53].

We chose to emulate the dual-fluorophore approach described above for our KLR construct. The final vector that we produced for the KLR reporter encoded a single messenger RNA containing the following components: the KLR reporter, the T2A self-cleaving peptide, and an mRuby3 red fluorophore [76] linked to an NLS derived from simian virus 40 (SV40 NLS) [77]. If the resulting KLR reporter construct performs as expected, mRuby3 will be constitutively localized to the nuclei of plant cells due to the SV40 NLS, whereas the localization of the mNeonGreen-tagged KLR reporter should shift between the nucleus and the cytoplasm, depending on the phosphorylation status of the reporter (Figure 3B).

Upon creation of our KLR construct, the first step in our validation process was to determine if the KLR reporter could localize to or be excluded from plant cell nuclei in a phosphorylation-dependent manner. To investigate this, the serine phosphorylation sites in the bNLS domain of KLR were mutagenized to either the non-phosphorylatable alanine, creating a “phosphodead” variant (KLR^AA^), or the negatively-charged glutamic acid, resulting in a “phosphomimetic” construct (KLR^EE^). As KLR^AA^ mimicked a fully “unphosphorylated” biosensor, we expected it to localize mainly to the nucleus. In contrast, the fully “phosphorylated” KLR^EE^ was expected to localize mainly to the cytoplasm. Stable transgenic lines expressing KLR, KLR^AA^, or KLR^EE^ were produced and imaged using confocal microscopy. The fluorescence emission intensities of the mNeonGreen and mRuby3 fluorophores were captured for these samples. As described above, mNeonGreen fluorescence is derived from the KLR reporter, whereas mRuby3 red fluorescence serves as a nuclear marker in these experiments (Figure 3C). In most plant cells, the vacuole occupies much of the volume, compressing the cytoplasm and making quantification of the cytoplasmic fluorescent signal difficult. As such, we choose to image the root tips of seedlings expressing the KLR constructs, due to their relatively smaller vacuoles and the consequent ease of measuring cytoplasmic versus nuclear fluorescence.

Strong nuclear mNeonGreen fluorescence was observed in root cells that expressed either KLR or KLR^AA^ (Figure 3C). In contrast, root cells expressing KLR^EE^ had much lower mNeonGreen fluorescence in the nuclei, and higher mNeonGreen fluorescence in the cytoplasm (Figure 3C). The localization of KLR and KLR^AA^ appeared to be mainly nuclear, whereas that of KLR^EE^ was mainly cytoplasmic. When the ratio of cytoplasmic to nuclear mNeonGreen fluorescence intensity was calculated, it was significantly higher for KLR^EE^ than either KLR (*p* < 0.001, Student’s *t*-test) or KLR^AA^ (*p* < 0.001, Student’s *t*-test) (Figure 3D), indicating that cells expressing KLR^EE^ have more mNeonGreen fluorescence in the cytoplasm than those expressing KLR or KLR^AA^. These results are consistent with our expectation that the unphosphorylatable KLR^AA^ would localize mainly to the nuclei, whereas phosphomimetic KLR^EE^ would localize mainly to the cytoplasm.

### 2.4. Addition of a Plant MAPK Docking Domain to KLR Facilitates Subcellular Localization Shifts in Response to Chitin

The experiments described above using the KLR^EE^ construct containing phosphomimetic mutations suggested that the KLR reporter may have the potential to report kinase activity in plant cells. The KLR reporter, however, does not contain any kinase docking domain. In order to test KLR’s potential as plant MAPK reporter, it was therefore first necessary to add a plant MAPK docking domain to the KLR construct. For this experiment, we incorporated the MKP1 substrate domain from our previously described plant MAPK FRET biosensor SOMA [44,78] into KLR, creating KLR-MKP1 (Figure 3A). Since SOMA is able to report MAPK activity in living plant tissue, we reasoned that the MKP1 substrate domain must possess a functional plant MAPK docking domain. To minimize potentially confounding effects of the phosphosite present in this MKP1 substrate domain [44,66,67] we mutated that phosphorylation site to alanine so that the only phosphorylation sites present on the KLR-MKP1 construct were those present in the bNLS domain.

*Arabidopsis* lines stably expressing KLR-MKP1 were created and seedlings of these lines were placed in imaging chambers that allowed us to apply chemical treatments to the samples during imaging [79]. In order to determine if KLR-MKP1 had the potential to report in vivo changes in MAPK activation, we subjected the seedlings to treatment with an aqueous suspension of chitin, which is known to elicit the rapid activation of MPK3/6 in *Arabidopsis* [15,80,81]. Root tip cells of these seedlings were imaged using confocal microscopy to measure mNeonGreen and mRuby3 fluorescence. For each experiment, the first image was taken prior to chitin treatment, while the seedling was resting in pure water. After this image was taken, the water within the imaging chamber was replaced with an aqueous suspension of chitin, and two additional images were collected at time points 12 min and 52 min following chitin treatment.

When the three time points of the mNeonGreen channel were examined, we observed weaker nuclear fluorescence and stronger cytoplasmic fluorescence at the 12 min time point in comparison to the 0 min and 52 min time points. In contrast, there were no substantial differences between the three time points for the mRuby3 channel (Figure 3E). When the fluorescent emission intensity was quantified, the nuclear mNeonGreen signal at the 12 min mark had decreased by ca. 60% when compared to the 0 min time point, whereas the cytoplasmic mNeonGreen fluorescent intensity had increased by ca. 61% (Figure 3G). In contrast, the difference in nuclear and cytoplasmic mNeonGreen fluorescent intensities at the 52 min time point to those of the 0 min time point were ca. 0% and ca. 5%, respectively (Figure 3G). These data are consistent with a model in which the KLR-MKP1 reporter transiently leaves the nucleus for the cytoplasm in response to chitin treatment.

As a control, we also tested the response to chitin of the KLR reporter lacking a MAPK docking domain. In these experiments, chitin treatment had no effect on the nuclear localization of KLR (Supplementary Figure S2A–C). This result is consistent with the hypothesis that a MAPK docking domain is required for KLR to change its subcellular localization in response to treatments known to activate MAPK activity in *Arabidopsis*.

Based on these results, the addition of the MKP1 substrate domain to KLR resulted in a reporter that appears to have changed localization in response to chitin treatment in *Arabidopsis* seedlings. To determine if this apparent change in localization was dependent on the bNLS phosphorylation sites present in KLR, the phosphosites were mutagenized to non-phosphorylatable alanine residues, resulting in a “phosphodead” KLR^AA^-MKP1 reporter. When seedlings expressing KLR^AA^-MKP1 were treated with chitin, no substantial changes in either mNeonGreen or mRuby3 fluorescent signal were observed (Figure 3F,H). These results are consistent with a model in which the nucleocytoplasmic shuttling of KLR-MKP1 is regulated by its phosphorylation sites, likely through their phosphorylation.

### 2.5. KLR-MKP1 Exhibited Shifts in Localization in Arabidopsis Cotyledons in Response to Different Chemical Treatments Known to Trigger MAPK Activation

**Figure 4:**
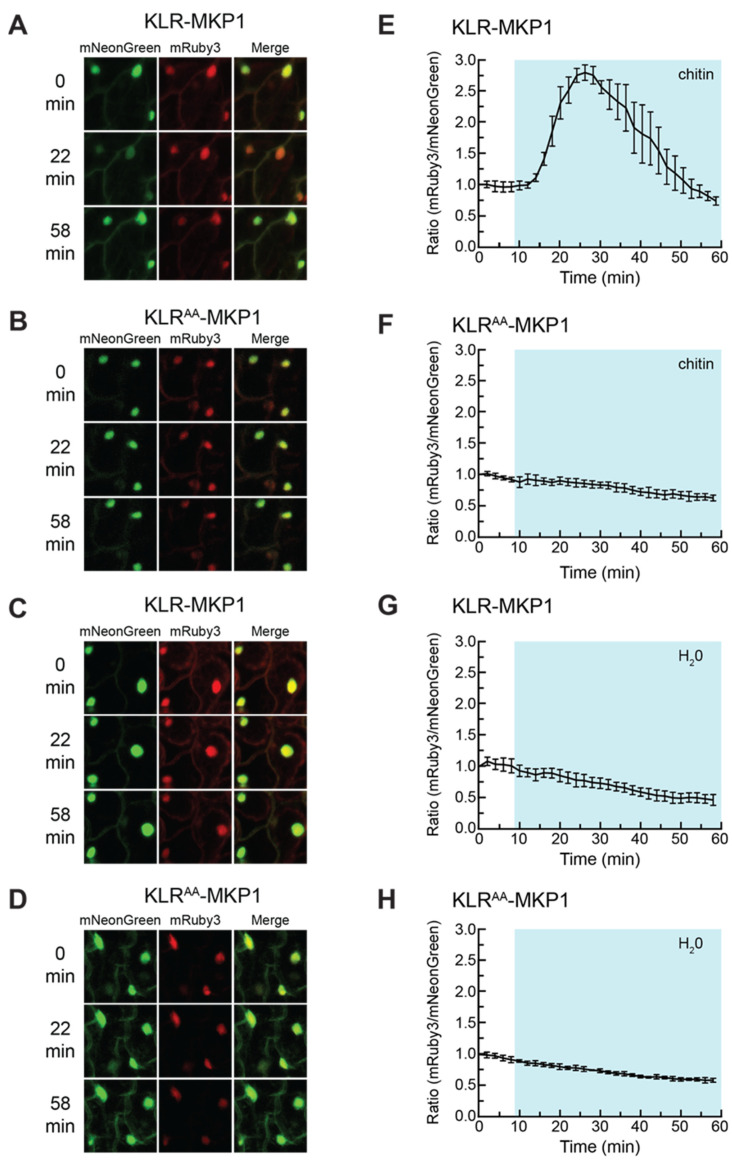
Chitin causes a transient reduction in nuclear mNeonGreen fluorescence of KLR-MKP1. (**A**–**D**) *Arabidopsis* cotyledons expressing KLR-MKP1 or KLR^AA^-MKP1 were treated with chitin (**A**,**B**) or water (**C**,**D**) and imaged every two minutes using confocal microscopy at 20× optical magnification. The mNeonGreen, mRuby3, and merged channels for the 0 min, 22 min, and 58 min time points are shown for each experiment. mNeonGreen emission is shown in green. mRuby3 emission is shown in red. (**E**–**H**) Quantification of the nuclear fluorescent emission for the experiments shown in (**A**–**D**). Each data point represents the ratio of the average mNeonGreen and mRuby3 emission intensities within four regions of interest (ROIs) corresponding to cell nuclei. The data were normalized by dividing each value in the time-course by the 0 min value. The shaded background on each graph indicates when the sample was exposed to a given treatment. During the first 10 min of each experiment the samples were incubated in pure water. Error bars indicate standard deviation. Videos are available as Movies S1–S4.

To further explore the potential of KLR-MKP1 as a MAPK biosensor, we tested the behavior of KLR-MKP1 under experimental conditions that had been previously used to characterize the *Arabidopsis* MAPK FRET biosensor SOMA [44]. *Arabidopsis* cotyledons expressing KLR-MKP1 were imaged every 2 min, with chitin added between the 8 min and 10 min time points (Figure 4A). As cytoplasmic mNeonGreen fluorescence was difficult to quantify in this tissue type, fluorescent emission intensity was measured solely in the nuclei of cells. In response to chitin, the nuclear mNeonGreen signal of KLR-MKP1 dropped ca. 60% before returning to pre-treatment levels at the 52 min time point, whereas nuclear mRuby3 exhibited no such change (Supplementary Figure S3A).

As we were unable to accurately quantify the cytoplasmic mNeonGreen signal, we normalized the data by dividing the nuclear mRuby3 fluorescence intensity by the nuclear mNeonGreen fluorescent intensity (Figure 4E), following the convention established by the developers of the nKTR reporter [53]. Normalizing the data in this way produces numerical results in which an increase in kinase activity is expected to result in an increase in the nuclear mRuby3/mNeonGreen ratio. When this approach was employed, the resulting ratio of nuclear mRuby3/mNeonGreen fluorescence intensity rapidly increases upon exposure to chitin, reaching a peak gain of ca. 180% before returning to basal levels 42 min after chitin was added. The increase in this ratio is driven by dramatic reductions in the nuclear mNeonGreen signal, whereas the nuclear mRuby3 signal does not undergo large changes (Supplementary Figure S3A).

We also repeated this experiment using the non-phosphorylatable KLR^AA^-MKP1 as a control. When cotyledons expressing KLR^AA^-MKP1 were treated with chitin (Figure 4B), there was no increase in the mRuby3/mNeonGreen ratio (Figure 4F), and the nuclear mNeonGreen fluorescence signal did not display any dramatic changes (Supplementary Figure S3B). The observation that KLR^AA^-MKP1 does not change localization in response to chitin treatment is consistent with our hypothesis that phosphorylation of the bNLS is responsible for the changes in nuclear fluorescence observed with KLR-MKP1. As additional controls, cotyledons expressing either construct were also treated with pure water rather than the aqueous chitin solution, (Figure 4C,D,G,H, and Figure S3C,D) or imaged with no additional manipulation (Figure S4A–F). No substantial responses were observed for these experiments, demonstrating that the imaging protocol was not responsible for the changes in nuclear mNeonGreen fluorescence observed when cotyledons expressing KLR-MKP1 were exposed to chitin.

**Figure 5:**
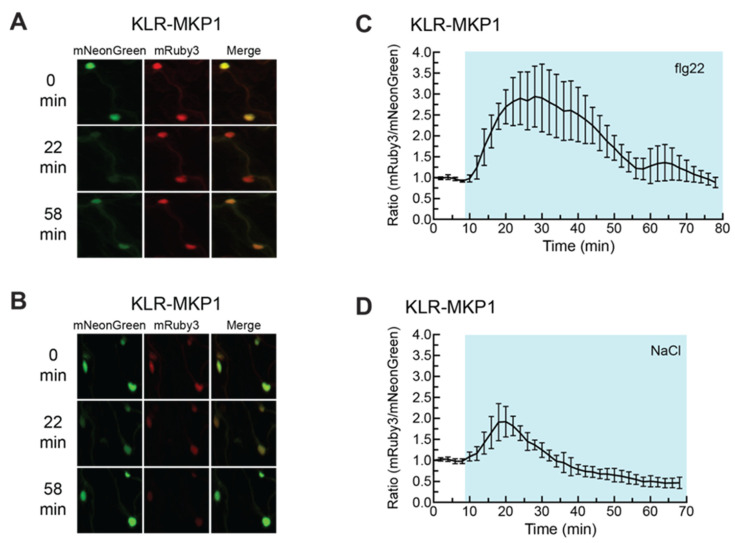
flg22 and NaCl cause a transient reduction in nuclear mNeonGreen fluorescence of KLR-MKP1. (**A**,**B**) *Arabidopsis* cotyledons expressing KLR-MKP1 were treated with 1µ M of flg22 (**A**) or 150 mM NaCl (**B**) and were imaged every two min using confocal microscopy at 20× optical magnification. The mNeonGreen, mRuby3, and merged channels for the 0 min, 22 min, and 58 min time points are shown for each experiment. mNeonGreen emission is shown in green. mRuby3 emission is shown in red. (**C**,**D**) Quantification of the nuclear fluorescent emission for the experiments shown in (**A**,**B**). Each data point represents the ratio of the average mNeonGreen and mRuby3 emission intensities within four regions of interest (ROIs) corresponding to cell nuclei. The data were normalized by dividing each value in the time-course by the 0 min value. The shaded background on each graph indicates when the sample was exposed to a given treatment. During the first 10 min of each experiment the samples were incubated in pure water. Error bars indicate standard deviation. Videos are available as Movies S5 and S6.

We next wanted to determine if KLR-MKP1 localization could be affected by additional chemical treatments know to activate MAPKs. Cotyledons expressing KLR-MKP1 were treated with either the synthetic flagellin peptide flg22 (Figure 5A,C and Figure S5A) or NaCl (Figure 5B,D, and Figure S5B). Both treatments have been previously demonstrated to trigger MAPK activation [8,10,11,14,82,83,84,85,86] and had also induced an increase in FRET efficiency for the plant MAPK FRET biosensor SOMA [44]. When cotyledons expressing KLR-MKP1 were exposed to a 1 µM solution of flg22, an initial increase in the nuclear mRuby3/mNeonGreen ratio of ca. 190% was observed, which lasted for 46 min (Figure 5C). This first peak was followed by a weaker second increase approximately 47 min after flg22 was added. When 150 mM NaCl was the treatment, the increase in nuclear mRuby3/mNeonGreen ratio was smaller, with a gain of ca. 92%, and more transient, lasting for only 26 min (Figure 5D). The observation that KLR-MKP1 displays different responses to chitin, flg22, and NaCl treatments raises the possibility that this type of biosensor can offer insights into the mechanisms by which these external stimuli can lead to differential activation of response pathways.

### 2.6. Incorporating the AP2C1 Docking Domain into KLR Produces a Biosensor That Changes Localization in Response to Chitin

The work described above with KLR-MKP1 suggested that KLR has the potential to serve as a kinase activity reporter in *Arabidopsis*, provided that a MAPK docking domain is added to the construct. To further test this hypothesis, we wanted to determine if adding a different MAPK docking domain to KLR would produce a biosensor that was able to respond to chitin treatment. For this experiment, we chose the AP2C1 docking domain that we characterized using the in vitro docking domain trap described above in Section 2.2. We chose this docking domain because it had driven the highest MAPK-induced increase in FRET efficiency in the in vitro assay. The KLR construct that we produced by adding the AP2C1 docking domain was named KLR-AP2C1. A “phosphodead” variant of this biosensor was also created by mutagenizing the bNLS phosphorylation sites into alanine to produce KLR^AA^-AP2C1.

**Figure 6:**
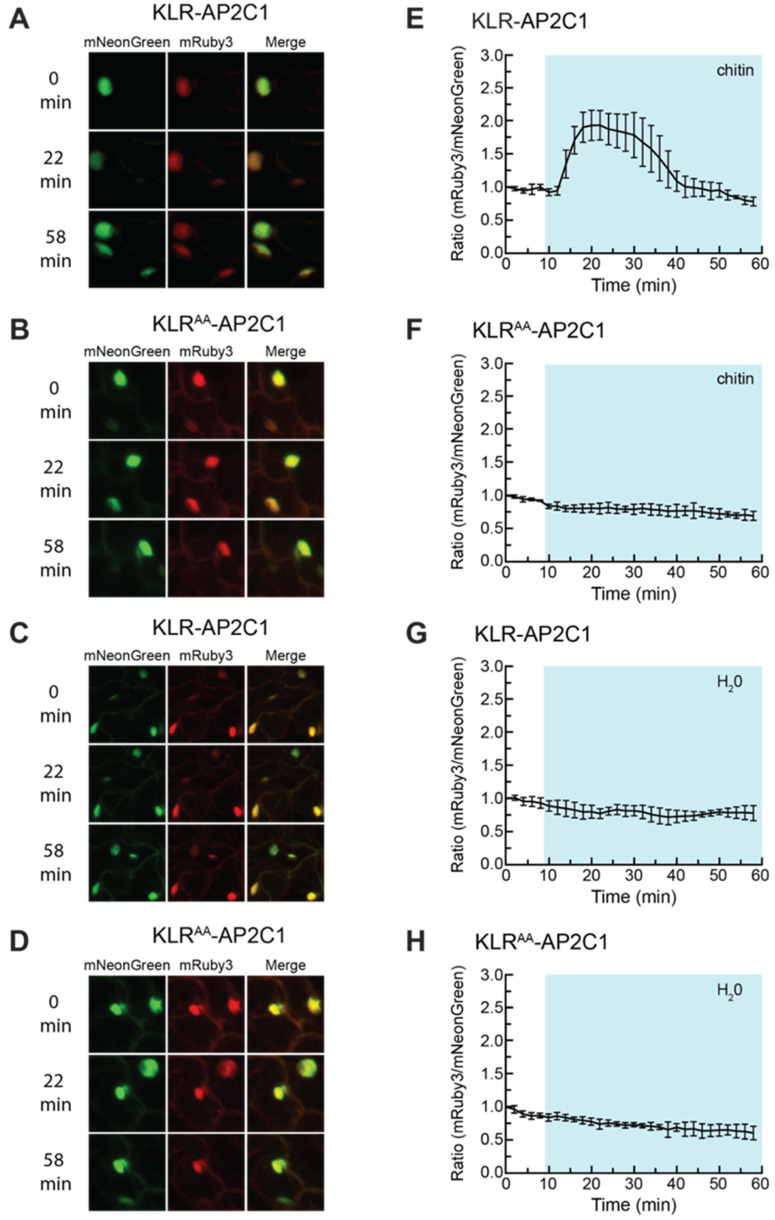
KLR-AP2C1 exhibits a transient reduction in nuclear mNeonGreen fluorescence in response to chitin treatment. (**A**–**D**) *Arabidopsis* cotyledons expressing KLR-AP2C1 or KLR^AA^-AP2C1 were treated with 40 mg/mL chitin (**A**,**B**) or 200 µL water (**C**,**D**) and imaged every two minutes using confocal microscopy at 20× optical magnification. The mNeonGreen, mRuby3, and merged channels for the 0 min, 22 min, and 58 min time points are shown for each experiment. mNeonGreen emission is shown in green. mRuby3 emission is shown in red. (**E**–**H**) Quantification of the nuclear fluorescent emission for the experiments shown in (**A**–**D**). Each data point represents the ratio of the average mNeonGreen and mRuby3 emission intensities within four regions of interest (ROIs) corresponding to cell nuclei. The data were normalized by dividing each value in the time-course by the 0 min value. The shaded background on each graph indicates when the sample was exposed to a given treatment. During the first 10 min of each experiment the samples were incubated in pure water. Error bars indicate standard deviation. Videos are available as Movies S7–S10.

Cotyledons from stable transgenic lines expressing either KLR-AP2C1 or KLR^AA^-AP2C1 were treated with chitin, pure water, or received no treatment (Figure 6A–H, Supplementary Figure S6A–D, Supplementary Figure S7A–F). The addition of chitin to cotyledons expressing KLR-AP2C1 triggered a ca. 193% increase in the nuclear mRuby3/mNeonGreen ratio over 32 min (Figure 6E), corresponding to a ca. 42% drop in nuclear mNeonGreen signal over the same period of time (Supplementary Figure S6A). As expected, pure water and no treatment did not induce a gain in the nuclear mRuby3/mNeonGreen ratio or a drop in nuclear mNeonGreen signal. These results demonstrated that a MAPK docking domain identified by the in vitro docking domain trap could be added to the KLR construct to produce a biosensor capable of exhibiting a shift in subcellular localization in response to a chemical elicitor known to induce MAPK activity.

## 3. Discussion

MAPKs play an essential role in mediating numerous cellular processes. In many signaling networks, activation of MAPKs is necessary to translate external stimuli into a specific biological response. To better understand the mechanisms by which MAPKs operate, quantification of their spatiotemporal dynamics would be advantageous. Genetically encoded biosensors serve as potential tools for capturing these kinetics, offering several advantages over biochemical methods for studying kinase activation. In animal models, fluorescent biosensors targeted to MAPKs have been widely used, while application of these tools to the field of plant MAPK research has lagged behind. To facilitate further adoption of MAPK fluorescent biosensors for plant research, we have established an experimental pipeline that utilizes both in vitro and in vivo components to efficiently streamline the process of developing new fluorescent biosensors for studying MAPKs in plants.

The first part of this paper describes an in vitro FRET-based assay for evaluating plant MAPK docking domains. This “docking domain trap” was developed by repurposing a FRET-based biosensor developed for studying the activation of MAPKs in animal cells called EKAREV [48]. EKAREV possesses a phosphoamino acid binding domain, phosphoacceptor domain, two fluorophores, and a linker that have all been shown to work effectively together to serve as a kinase activity biosensor. For application in plants, the only functional element missing from this construct is a MAPK docking domain that interacts effectively with plant MAPKs. Indeed, inserting known plant MAPK docking domains from the *Arabidopsis* proteins BASL or AP2C1 into the EKAREV backbone resulted in biosensors that underwent a 10-fold increase in their FRET efficiency when treated with constitutively active versions of the *Arabidopsis* MAPKs MPK4 or MPK6 when compared to the EKAREV backbone alone [62]. Furthermore, mutagenizing the phosphorylation site within the EKAREV phosphoacceptor for either construct abolished this increase in FRET, suggesting that the CA-MAPK isoforms induce an increase in FRET efficiency in a phosphorylation-dependent manner.

One of the potential applications of this plant MAPK docking domain trap would be the development of fluorescent biosensors that specifically report the activation of a single MAPK isoform. A systematic approach to accomplishing this goal would be to screen a large panel of candidate docking domains in vitro using the EKAREV plant MAPK docking domain trap to find candidate sequences that show promising activity profiles in response to a panel of constitutively active MAPK variants that can be effectively expressed as recombinant proteins. Site-directed mutagenesis or molecular evolution could then be employed to further refine these candidate sequences to ultimately produce a docking domain that possesses input specificity towards a MAPK isoform of interest. These docking domains could then be deployed in vivo as either FRET biosensors using the EKAREV backbone or as translocation biosensors using the approach described below. If such isoform-specific reporters can be developed, they should allow researchers to more precisely investigate issues related to specificity and cross-talk in plant signaling pathways.

In addition to the docking domain trap described above, the other main focus of our study was to expand the types of fluorescent kinase biosensors currently available to plant researchers. For this work we focused on translocation biosensors, which are a type of kinase reporter that changes its subcellular localization in response to phosphorylation of a bNLS/NES module present within the biosensor [51]. Starting with the nKTR reporter developed for measuring kinase activity in *C. elegans* [53], we removed the 44 amino acid Elk1 docking domain and optimized the coding sequence for expression in plants, naming the resulting construct KLR, for kinase localization reporter. To investigate whether KLR localization was influenced by the phosphorylation status of the bNLS/NES domain, we created a “phosphodead” KLR^AA^ variant and a “phosphomimetic” KLR^EE^ variant in which the two phosphorylation sites were mutated to either alanine or glutamic acid residues. KLR^AA^ was localized largely in the nucleus, whereas the phosphomimetic KLR^EE^ was localized largely in the cytoplasm. These results suggested that the phosphorylation-responsive bNLS/NES developed for animal cells was functional in plant cells. Follow-up experiments, in which we added plant MAPK docking domains to our KLR construct, provided further evidence that KLR has the capacity to serve as a kinase reporter in plants. Specifically, we observed that KLR variants with plant MAPK docking domains showed transient reductions in nuclear fluorescence emission intensity when plant tissue expressing these reporters was exposed to chitin, flg22, or NaCl, which are treatments known to activate MAP kinases in plants.

Our observation that the nuclear fluorescence intensities of KLR-MKP1 and KLR-AP2C1 undergo transient reductions in response to stress treatments known to activate MAPKs is consistent with the hypothesis that these biosensors are reporting the activity of MAPKs in vivo. Further experimentation will be needed, however, to prove this hypothesis. Our observation that the phosphorylation sites within the bNLS/NES of KLR are necessary for the stress-induced changes in nuclear fluorescence intensity is consistent with the idea that phosphorylation of the NLS/NES by a kinase is responsible for the changes in fluorescent signal observed. In order to prove that a MAPK is responsible for this in vivo phosphorylation, it will be necessary to generate transgenic lines that express the KLR-MKP1 or KLR-AP2C1 reporter in a genetic background carrying null mutants of one or more *Arabidopsis* MAPKs. Because many MAPK substrates in *Arabidopsis* are able to be phosphorylated by multiple MAPK isoforms [25], it may be necessary to work with mutant lines that have more than one member of the MAPK gene family knocked out. This approach is complicated by the fact that MPK3/6, the two MAPKs known to interact with MKP1 and AP2C1, are a synthetic lethal gene pair [87]. In our previous work with the plant MAPK FRET biosensor SOMA, we demonstrated how the use of an analog-sensitive variant of MPK6 expressed in a *mpk3/6*-null background can enable one to use a chemical inhibitor to specifically switch off MPK6 activity [44,88,89]. Using that approach, we were able to demonstrate that the SOMA biosensor was reporting MPK6 activation in response to NaCl treatment. A similar approach could be taken to directly test if KLR-MKP1 or KLR-AP2C1 are acting as MAPK biosensors in *Arabidopsis.*

## 4. Materials and Methods

### 4.1. Plasmid Construction

EKAREV was a kind gift from Michiyuki Matsuda and Kazuhiro Aoki. Specifically, we used the previously described SOMA construct [44] and replaced the substrate domain encoding amino acid residues 15–94 of the *Arabidopsis* MKP1 protein with the 18 amino acids that make up the optimized substrate sequence in the EKAREV construct, including the FQFP kinase docking domain, [48] using restriction enzyme cloning. To create EKAREV-BASL, a region encoding amino acid residues 51–89 of the *Arabidopsis* BASL protein was added to the EKAREV construct. Additionally, the serine at position 72 of the BASL protein was mutagenized to an alanine. To create EKAREV-AP2C1, a region encoding amino acid residues 92–124 of the *Arabidopsis* AP2C1 protein was added to the EKAREV construct. To create the phosphorylation site mutant forms of the above EKAREV variants, site-directed mutagenesis was performed to change the coding sequence at the threonine phosphorylation site of the sensor protein to alanine. The resulting protein-coding sequences were then cloned into a derivative of Pet-32(a)+ (Millipore Sigma, (https://www.emdmillipore.com/) for expression of recombinant proteins in *E. coli*. Plasmid maps and DNA sequence for pET-EKAREV, pET-EKAREV-BASL, pET-EKAREV^T48A^-BASL, pET-EKAREV-AP2C1, and pET-EKAREV^T48A^-AP2C1 are provided in Data Files S1–S5. The full plasmid maps for the pKLR expression vector and its derivatives pKLR^AA^, pKLR^EE^, pKLR-AP2C1, pKLR^AA^-AP2C1, pKLR-MKP1, and pKLR^AA^-MKP1 are provided in Data Files S6–S12.

### 4.2. In Vitro FRET Assay

The in vitro FRET assay was performed as previously described [44]. Recombinant EKAREV, SOMA, EKAREV-BASL, EKAREV^T48A^-BASL, SOMA^T679A^, EKAREV-AP2C1, EKAREV^T48A^-AP2C1, CA-MPK6, and CA-MPK4 were expressed in the Rosetta2 strain of *E. coli* (Millipore Sigma, Temecula, CA, USA). CA-MPK6 and CA-MPK4 are constitutively active forms of *Arabidopsis* MPK6 and MPK4, respectively [62]. Plasmids expressing CA-MPK6 or CA-MKP4 were kind gifts from Jean Colcombet. To perform the assay, Rosetta2 *E. coli* cells were grown overnight in 1 mL lysogeny broth (LB) with the appropriate antibiotics at 25 °C at 276 revolutions per min (RPM). The next day, 200 µL of each culture was added to fresh tubes of 1 mL LB containing the appropriate antibiotics and 0.1 mM Isopropyl β-D-1-thiogalactopyranoside (IPTG) (Sigma-Aldrich), before growing overnight at 25 °C at 276 RPM. The next day, the 1.2 mL cultures were spun down, supernatant was removed, and the tubes were incubated at −20 °C for at least 30 min. Tubes were then returned to room temperature and the cell pellets was treated with 200 µL Bug Buster reagent (Millipore Sigma) for the *E. coli* expressing biosensor proteins or 100 µL Bug Buster for the *E. coli* expressing CA-MPK6 or CA-MPK4. Samples were then centrifuged at 4 °C at 13,000 rpm in a microcentrifuge and the cleared lysate was transferred to new tubes. Seven microliters of the cleared lysate containing recombinant biosensor protein was added to a reaction containing 25 mM 2-amino-2-(hydroxymethyl)-1,3-propanediol (TRIS)-HCl, 0.5 mM DTT, 5 mM MgCl2, 0.1 mM ATP, 100 mM NaCl and water to a total volume of 200 µL. For reactions including CA-MPK6 or CA-MPK4, 4 µL of the appropriate CA-MAPK cleared lysate was also added. The reactions were then incubated at room temperature for 30–60 min and the fluorescent emission spectrum was measured using a QuantaMaster 40 Spectrofluorometer. Excitation was performed at 420 nm, and the emission range analyzed was 465–545 nm. The slit size for both excitation and emission was 5 nm with a step size of 0.5 nm and integration of 0.1 nm. All experiments were performed with at least three technical reps.

### 4.3. Transgenic Lines

Transgenic lines expressing KLR, KLR^AA^, KLR^EE^, KLR-MKP1, KLR^AA^-MKP1, KLR-AP2C1, and KLR^AA^-AP2C1 were produced via the floral dip method using the GV3101 strain of *Agrobacterium tumefaciens* [90]. Primary transformants expressing the biosensor constructs were identified by screening seedlings with an epifluorescence microscope. These seedlings were 4-day-old seedlings germinated on 1% agar (*w/v*) plates containing 0.5× Murashige and Skoog basal salt mixture. Seedlings exhibiting strong fluorescence were transferred to soil and seed was collected. Seed for the transgenic lines is available via the Arabidopsis Biological Resource Center (ABRC) (http://abrc.osu.edu/).

### 4.4. Plant Material and Growth Conditions

To prepare seedlings for confocal imaging, seeds were surface sterilized using 95% ethanol and then plated onto growth media composed of 1% agar (*w*/*v*) containing 0.5× Murashige and Skoog basal salt mixture in 130 mm square Petri dishes. The samples were incubated in the dark at 4 °C for 2 days to stratify the seeds and then placed under constant light at 20 °C–25 °C with the plates in a vertical orientation. Seedlings were collected for imaging after the plates had been under light for 3–4 days. Cotyledons were collected for imaging after the plates had been under light for 4–5 days.

### 4.5. Confocal Microscopy

Whole seedlings or detached cotyledons were prepared for imaging on the confocal microscope using the previously described HybriWell^TM^ method [79]. Briefly, a 5 µL drop of ultrapure water was placed on a 45 mm × 55 mm microscope cover glass. Then, the sample was placed on top of the droplet (cotyledons were place abaxial side down). The sample was gently submerged by dripping water around it with a pipette. Excess water was then removed, and a HybriWell^TM^ (Grace Bio-Labs, http://gracebio.com/, cat. no. 611102) was gently placed on the cover slide with the sample centered to form a 150 µm-deep imaging chamber with a volume of 30 µL. The dimensions of the chamber are such that the sample is gently held in place by pressure from the top of the chamber, but liquid can still pass between the sample and the cover glass [79]. Then, 400 µL of ultrapure water was injected through one of the HybriWell^TM^ ports using a pipettor to fill the 30 µL chamber with water and expel any air bubbles. A 200 µL droplet of ultrapure water was then placed on one of the ports to prevent the chamber from drying out. The HybriWells^TM^ were then placed in covered Petri dishes and equilibrated by incubating at 20–23 °C under constant light for 12–16 h prior to imaging.

Confocal microscopy was performed using a Zeiss LSM 780 (http://www.zeiss.com/) with a 40× objective for root tips or a 20× objective for cotyledons. mNeonGreen was excited at 458 nm at 2% power and mRuby3 was excited at 561 nm at 2.5% power. Emission was measured between 489 nm and 544 nm for mNeonGreen and between 579 nm and 632 nm for mRuby3. Z-stacks were collected every 2 min with an optical slice thickness of 2 µm.

Chemical treatments were added to the samples during imaging by pipetting 200 µL of solution containing the treatment onto one port of the HybriWell^TM^. For chitin treatment, 200 mg of chitin from shrimp shells (Sigma, http://www.sigmaaldrich.com/, cat. no. C7170–100G) was added to a 1.5 mL Eppendorf tube containing two steel pellets and ground at 25 Hz for 10 min using a Retsch MM 200 mixer mill (Retsch, http://www.retsch.com/). The pulverized chitin was then added to 5 mL of pure water and vortexed briefly prior to use, for a working concentration of 40 mg/mL. For flg22 treatment, a 1 µM solution of synthetic flg22 peptide (QRLSTGSRINSAKDDAAGLQIA) (PhytoTechnology Laboratories, http://phytotechlab.com/, cat. no. P6622) in 5 mL pure water was used.

### 4.6. Image Analysis

Post-processing of the raw image data was performed using Fiji [91]. Image sets were concatenated before the ‘Z-projection’ function was performed on an image stack using the ‘Max Intensity’ setting. To quantify the emission channels individually, the ‘Subtract Background’ function was performed, with the ‘rolling ball radius’ set at the default 50 pixels. The resulting image stack was then converted to 32-bit, and the ‘Threshold’ function was performed with the default values by selecting ‘Apply’ and enabling the ‘Set to NaN’ option. To create a merged image, the channels were split and then merged back together. Movies S11, S13–S16, S19–S26, S29, and S30 present time-course videos of the merged image data for select experiments.

To create a ratio image, the Z-projection was divided into separate mNeonGreen and mRuby3 emission channels. The ‘Subtract Background’ function was performed on both images, with the ‘rolling ball radius’ set as the default 50 pixels. A mask was then created from the mRuby3 channel using the ‘Convert to Mask’ function. The background-subtracted mRuby3 and mNeonGreen images were then converted into 32-bit images. These 32-bit images were then multiplied by the Mask file. The resulting mRuby3 image was divided by the resulting mNeonGreen image using the ‘Image Calculator’ function to create a ratio image representing the ratio of mRuby3 to mNeonGreen emission. Finally, the ‘Threshold’ function was performed using the default values, with the ‘NaN background’ option enabled. The ‘Fire’ lookup table was then applied to the final ratio image. To measure the ratio of mRuby3 to mNeonGreen emission, a region of interest (ROI) containing a nucleus was manually selected using Fiji and then average intensity was measured. For time-course experiments, the change in the mRuby3 to mNeonGreen emission ratio was normalized to the initial value of the mRuby3/mNeonGreen emission ratio and plotted versus time. Movies S1–S10, S12, S17, S18, S27, and S28 present time-course videos of the ratio image data for selected experiments.

## 5. Conclusions

In this study we have described an experimental pipeline for building new FRET and translocation biosensors as complimentary tools to study MAPK signaling in *Arabidopsis*. The first component of this pipeline is an in vitro FRET-based assay for identifying and assessing the specificity of plant MAPK docking domains. Docking domains that perform well in this in vitro assay can remain within the EKAREV construct to be directly used as MAPK FRET biosensors in plants or swapped into the KLR translocation biosensor construct for use in vivo. It is our hope that this pipeline will allow the diversity of plant MAPK fluorescent biosensors to continue to grow and broaden, providing the research community with additional tools to further document and explore the role MAPKs play in the regulation of signaling pathways in plants.

## Figures and Tables

**Table 1:** In vitro FRET of EKAREV and SOMA variants.

	No Kinase			CA-MPK6				CA-MPK4			
	Turq	YPet	YPet/Turq	Turq	YPet	Turq	Δ FRET	Turq	YPet	Turq	Δ FRET
EKAREV	102,547	76,365	0.74	106,420	86,561	0.81	9%	111,014	82,988	0.75	0%
SOMA	77,325	69,725	0.90	63,494	96,300	1.52	68%	73,668	84,687	1.15	27%
EKAREV-BASL	81,070	58,495	0.72	56,506	87,706	1.55	115%	62,386	83,222	1.33	85%
EKAREV^T48A^-BASL	75,400	55,327	0.73	84,693	60,990	0.72	−2%	83,782	59,807	0.71	−3%
SOMA^T679A^	65,166	60,931	0.94	71,900	65,530	0.91	−3%	67,915	62,828	0.93	−1%
EKAREV-AP2C1	89,861	66,267	0.74	55,116	101,647	1.84	150%	55,971	99,090	1.77	140%
EKAREV^T48A^-AP2C1	87,272	66,980	0.77	97,788	74,682	0.76	0%	95,430	73,391	0.77	0%

Rows indicate specific biosensor variants. ‘No Kinase’ indicates no kinase was added to the extract containing the biosensor protein when fluorescent emission intensity was measured. ‘CA-MPK6′ indicates constitutively active MPK6 was present. ‘CA-MPK4′ indicates constitutively active MPK4 was present. ‘Turq’ is the average value of the three highest sequential data points for the Turquoise GL emission peak. ‘YPet’ is the average value of the three highest sequential data points for the YPet emission peak. ‘YPet/Turq’ is YPet divided by Turq. ‘ΔFRET’ was calculated by dividing the YPet/Turq ratio with No Kinase by either the YPet/Turq ratio plus CA-MPK6 or CA-MPK4.

## Supplementary Materials

Supplementary materials can be found at https://www.mdpi.com/1422-0067/21/15/5350/s1. Figure S1. SOMAT679A does not exhibit an increase in FRET efficiency in response to either CA-MPK6 or CA-MPK4. Figure S2. KLR without a plant docking domain does not exhibit a transient reduction in nuclear mNeonGreen fluorescence in response to chitin. Figure S3. KLR-MKP1 exhibits a transient reduction in nuclear mNeonGreen fluorescence in response to chitin. Figure S4. Behavior of KLR-MKP1 in the absence of a treatment. Figure S5. KLR-MKP1 exhibits a transient reduction in nuclear mNeonGreen signal in response to flg22 and NaCl. Figure S6. KLR-AP2C1 exhibits a transient decrease in nuclear mNeonGreen signal in response to chitin. Figure S7. Behavior of KLR-AP2C1 in the absence of a treatment. Movie S1. Figure 4A: KLR-MKP1 chitin. Movie S2. Figure 4B: KLRAA-MKP1 chitin. Movie S3. Figure 4C: KLR-MKP1 pure water. Movie S4. Figure 4D: KLRAA-MKP1 pure water. Movie S5. Figure 5A: KLR-MKP1 flg22. Movie S6. Figure 5B: KLR-MKP1 NaCl. Movie S7. Figure 6A: KLR-AP2C1 chitin. Movie S8. Figure 6B: KLRAA-AP2C1 chitin. Movie S9. Figure 6C: KLR-AP2C1 pure water. Movie S10. Figure 6D: KLRAA-AP2C1 pure water. Movie S11. Figure S2A: KLR-B chitin. Movie S12. Figure S2B: KLR-B chitin. Movie S13. Figure S3A: KLR-MKP1 chitin. Movie S14. Figure S3B: KLRAA-MKP1 chitin. Movie S15. Figure S3C: KLR-MKP1 pure water. Movie S16. Figure S3D: KLRAA-MKP1 pure water. Movie S17. Figure S4A: KLR-MKP1. Movie S18. Figure S4B: KLRAA-MKP1. Movie S19. Figure S4C: KLR-MKP1. Movie S20. Figure S4D: KLRAA-MKP1. Movie S21. Figure S5A: KLR-MKP1 flg22. Movie S22. Figure S5B: KLR-MKP1 NaCl. Movie S23. Figure S6A: KLR-AP2C1 chitin. Movie S24. Supplementary Figure S6B: KLRAA-AP2C1 chitin. Movie S25. Figure S6C: KLR-AP2C1 pure water. Movie S26. Figure S6D: KLRAA-AP2C1 pure water. Movie S27. Figure S7A: KLR-AP2C1. Movie S28. Figure S7B: KLRAA-AP2C1. Movie S29. Figure S7C: KLR-AP2C1. Movie S30. Figure S7D: KLRAA-AP2C1. Data S1. Plasmid map and DNA sequence of pET-EKAREV. Data S2. Plasmid map and DNA sequence of pET-EKAREV-BASL. Data S3. Plasmid map and DNA sequence of pET-EKAREVT48A-BASL. Data S4. Plasmid map and DNA sequence of pET-EKAREV-AP2C. Data S5. Plasmid map and DNA sequence of pET-EKAREVT48A-AP2C. Data S6. Plasmid map and DNA sequence of pKLR. Data S7. Plasmid map and DNA sequence of pKLRAA. Data S8. Plasmid map and DNA sequence of pKLREE. Data S9. Plasmid map and DNA sequence of pKLR-AP2C1. Data S10. Plasmid map and DNA sequence of pKLRAA-AP2C1. Data S11. Plasmid map and DNA sequence of pKLR-MKP1. Data S12. Plasmid map and DNA sequence of pKLRAA-MKP1.

## Abbreviations

MAPK	Mitogen-activated protein kinase
FRET	Förster resonance energy transfer
SOMA	Sensor of MAPK in *Arabidopsis*
NLS	Nuclear localization signal
NES	Nuclear exclusion signal
ERK	Extracellular signal-regulated kinases
MKP1	MAPK phosphotase 1
CA-MPK6	Constitutively active MAPK 6
CA-MKP4	Constitutively active MAPK 4
ATP	Adenosine triphosphate
BASL	Breaking of asymmetry in the stomatal lineage
AP2C1	*Arabidopsis* PP2C-type phosphatase
KTR	Kinase translocation reporter
bNLS	Bipartite nuclear localization signal
nKTR	Nuclear kinase translocation reporter
KLR	Kinase translocation reporter
T2A	*Thosea asigna* virus 2A peptide
SV40NLS	Simian virus 40 nuclear localization signal
ROI	Region of interest
Flg22	Flagellin 22
