# Supplementary material for: Expanding the Toolkit of Fluorescent Biosensors for Studying Mitogen Activated Protein Kinases in Plants
Source: Int J Mol Sci. 2020 Jul 28;21(15):5350. doi: 10.3390/ijms21155350 (PMC7432370; doi:10.3390/ijms21155350)
Supplement: Supplementary file 1 [file ijms-21-05350-s001.zip › Supp Fig Legends and File List.docx]

**SUPPLEMENTARY FIGURE LEGENDS**

**Supplementary Figure 1.** SOMA^T679A^ does not exhibit an increase in FRET efficiency in response to either CA-MPK6 or CA-MPK4. **(A,B)** Data from *in vitro* FRET assays measuring the emission spectrum of SOMA^T679A^ in response to excitation with 435 nm light in the presence or absence of constitutively active MPK6 (CA-MPK6) **(A)** or MPK4 (CA-MPK4) **(B)**. Three technical replications were performed for each experiment. Absence of a CA-MAPK is represented in blue. Presence of CA-MPK6 is represented in green. Presence of CA-MPK4 is represented in orange. Emission Intensity units are arbitrary “counts per second”. Error bars indicate standard deviation.

**Supplementary Figure 2.** KLR without a plant docking domain does not exhibit a transient reduction in nuclear mNeonGreen fluorescence in response to chitin. **(A)** Arabidopsis cotyledons expressing KLR (which lacks a plant MAPK docking domain) were treated with 200 mg/ml of chitin and imaged every two min using confocal microscopy. The mNeonGreen, mRuby3, and Merged channels for the 0 min, 22 min and 58 min time points are shown. **(B,C)** Quantification of the nuclear fluorescent emission for the experiment shown in **(A)**. In **(B)** each data point represents the ratio of the average mNeonGreen and mRuby3 emission intensities within four regions of interest (ROIs) corresponding to cell nuclei. In **(C)** each data point represents the average of the mNeonGreen or mRuby3 emission intensities within four regions of interest (ROIs) corresponding to cell nuclei. For **(B,C)** the data was normalized by dividing each value in the time-course by the 0 min value. The shaded background on each graph indicates when the sample was exposed to a given treatment. During the first 10 min of each experiment the samples were incubated in pure water. Error bars indicate standard deviation. Videos are available as Movies S11 and S12.

**Supplementary Figure 3.** KLR-MKP1 exhibits a transient reduction in nuclear mNeonGreen fluorescence in response to chitin. The data presented in this figure is a different representation of the same measurements shown in Figure 4. The same ROIs used to collect the quantitative image data in Figure 4 were used for this figure. In this figure, each data point represents the average of the mNeonGreen or mRuby3 emission intensities within four regions of interest (ROIs) corresponding to cell nuclei. The data was normalized by dividing each value in the time-course by the 0 min value. The shaded background on each graph indicates when the sample was exposed to a given treatment. During the first 10 min of each experiment the samples were incubated in pure water. Error bars indicate standard deviation. Videos are available as Movies S13-S16.

**Supplementary Figure 4.** Behavior of KLR-MKP1 in the absence of a treatment. **(A,B)** Arabidopsis cotyledons expressing KLR-MKP1 or KLR^AA^-MKP1 were incubated in 200 µl pure water and imaged every two min using confocal microscopy. The mNeonGreen, mRuby3, and Merged channels for the 0 min, 22 min and 58 min time points are shown for each experiment. **(C-F)** Quantification of the nuclear fluorescent emission for the experiments shown in **(A,B)**. In **(C,D)** each data point represents the ratio of the average mNeonGreen and mRuby3 emission intensities within four regions of interest (ROIs) corresponding to cell nuclei. In **(E,F)** each data point represents the average of the mNeonGreen or mRuby3 emission intensities within four regions of interest (ROIs) corresponding to cell nuclei. In **(C-F)** the data was normalized by dividing each value in the time-course by the 0 min value. The shaded background on each graph indicates when the sample was exposed to a given treatment. During the first 10 min of each experiment the samples were incubated in pure water. Error bars indicate standard deviation. Videos are available as Movies S17-S20.

**Supplementary Figure 5.** KLR-MKP1 exhibits a transient reduction in nuclear mNeonGreen signal in response to flg22 and NaCl. The data presented in this figure is a different representation of the same measurements shown in Figure 5. The same ROIs used to collect the quantitative image data in Figure 5 were used for this figure. In this figure, each data point represents the average of the mNeonGreen or mRuby3 emission intensities within four regions of interest (ROIs) corresponding to cell nuclei. The data was normalized by dividing each value in the time-course by the 0 min value. The shaded background on each graph indicates when the sample was exposed to a given treatment. During the first 10 min of each experiment the samples were incubated in pure water. Error bars indicate standard deviation. Videos are available as Movies S21 and S22.

**Supplementary Figure 6.** KLR-AP2C1 exhibits a transient decrease in nuclear mNeonGreen signal in response to chitin. The data presented in this figure is a different representation of the same measurements shown in Figure 6. The same ROIs used to collect the quantitative image data in Figure 6 were used for this figure. In this figure, each data point represents the average of the mNeonGreen or mRuby3 emission intensities within four regions of interest (ROIs) corresponding to cell nuclei. The data was normalized by dividing each value in the time-course by the 0 min value. The shaded background on each graph indicates when the sample was exposed to a given treatment. During the first 10 min of each experiment the samples were incubated in pure water. Error bars indicate standard deviation. Videos are available as Movies S23-S26.

**Supplementary Figure 7.** Behavior of KLR-AP2C1 in the absence of a treatment. **(A,B)** Arabidopsis cotyledons expressing KLR-AP2C1 or KLR^AA^-AP2C1 were incubated in 200 l water and imaged every two min using confocal microscopy. The mNeonGreen, mRuby3, and Merged channels for the 0’, 22’ and 58’ time points are shown for each experiment. **(C-F)** Quantification of the nuclear fluorescent emission for the experiments shown in **(A,B)**. In **(C,D)** each data point represents the ratio of the average mNeonGreen and mRuby3 emission intensities within four regions of interest (ROIs) corresponding to cell nuclei. In **(E,F)** each data point represents the average of the mNeonGreen or mRuby3 emission intensities within four regions of interest (ROIs) corresponding to cell nuclei. In **(C-F)** the data was normalized by dividing each value in the time-course by the 0 min value. The shaded background on each graph indicates when the sample was exposed to a given treatment. During the first 10 min of each experiment the samples were incubated in pure water. Error bars indicate standard deviation. Videos are available as Movies S27-S30.

**LIST OF SUPPLEMENTARY DATA FILES**

**Data S1.**  Plasmid map and DNA sequence of pET-EKAREV

**Data S2.**  Plasmid map and DNA sequence of pET-EKAREV-BASL

**Data S3.**  Plasmid map and DNA sequence of pET-EKAREV^T48A^-BASL

**Data S4.** Plasmid map and DNA sequence of pET-EKAREV-AP2C

**Data S5.** Plasmid map and DNA sequence of pET-EKAREV^T48A^-AP2C

**Data S6.** Plasmid map and DNA sequence of pKLR

**Data S7.** Plasmid map and DNA sequence of pKLR^AA^

**Data S8.** Plasmid map and DNA sequence of pKLR^EE^

**Data S9**. Plasmid map and DNA sequence of pKLR-AP2C1

**Data S10.** Plasmid map and DNA sequence of pKLR^AA^-AP2C1

**Data S11.** Plasmid map and DNA sequence of pKLR-MKP1

**Data S12.** Plasmid map and DNA sequence of pKLR^AA^-MKP1

**LIST OF SUPPLEMENTARY MOVIES**

**Movie S1.** Figure 4(A): KLR-MKP1 chitin

**Movie S2**. Figure 4(B): KLR^AA^-MKP1 chitin

**Movie S3.** Figure 4(C): KLR-MKP1 pure water

**Movie S4.** Figure 4(D): KLR^AA^-MKP1 pure water

**Movie S5**. Figure 5(A): KLR-MKP1 flg22

**Movie S6**. Figure 5(B): KLR-MKP1 NaCl

**Movie S7.** Figure 6(A): KLR-AP2C1 chitin

**Movie S8.** Figure 6(B): KLR^AA^-AP2C1 chitin

**Movie S9.** Figure 6(C): KLR-AP2C1 pure water

**Movie S10**. Figure 6(D): KLR^AA^-AP2C1 pure water

**Movie S11**. Supplementary Figure 2(A): KLR-B chitin

**Movie S12.** Supplementary Figure 2(B): KLR-B chitin

**Movie S13**. Supplementary Figure 3(A): KLR-MKP1 chitin

**Movie S14**. Supplementary Figure 3(B): KLR^AA^-MKP1 chitin

**Movie S15**. Supplementary Figure 3(C): KLR-MKP1 pure water

**Movie S16**. Supplementary Figure 3(D): KLR^AA^-MKP1 pure water

**Movie S17**. Supplementary Figure 4(A): KLR-MKP1

**Movie S18**. Supplementary Figure 4(B): KLR^AA^-MKP1

**Movie S19**. Supplementary Figure 4(C): KLR-MKP1

**Movie S20**. Supplementary Figure 4(D): KLR^AA^-MKP1

**Movie S21**. Supplementary Figure 5(A): KLR-MKP1 flg22

**Movie S22**. Supplementary Figure 5(B): KLR-MKP1 NaCl

**Movie S23**. Supplementary Figure 6(A): KLR-AP2C1 chitin

**Movie S24**. Supplementary Figure 6(B): KLR^AA^-AP2C1 chitin

**Movie S25**. Supplementary Figure 6(C): KLR-AP2C1 pure water

**Movie S26**. Supplementary Figure 6(D): KLR^AA^-AP2C1 pure water

**Movie S27**. Supplementary Figure 7(A): KLR-AP2C1

**Movie S28**. Supplementary Figure 7(B): KLR^AA^-AP2C1

**Movie S29**. Supplementary Figure 7(C): KLR-AP2C1

**Movie S30**. Supplementary Figure 7(D): KLR^AA^-AP2C1
