# Supplementary figures and images for: Expanding the Toolkit of Fluorescent Biosensors for Studying Mitogen Activated Protein Kinases in Plants
Source: Int J Mol Sci. 2020 Jul 28;21(15):5350. doi: 10.3390/ijms21155350 (PMC7432370; doi:10.3390/ijms21155350)

**A**SOMA<sup>T679A</sup>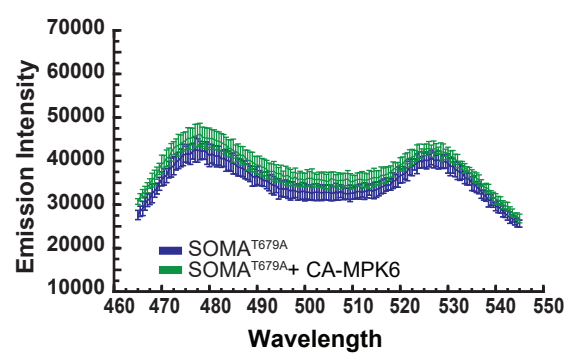**B**SOMA<sup>T679A</sup>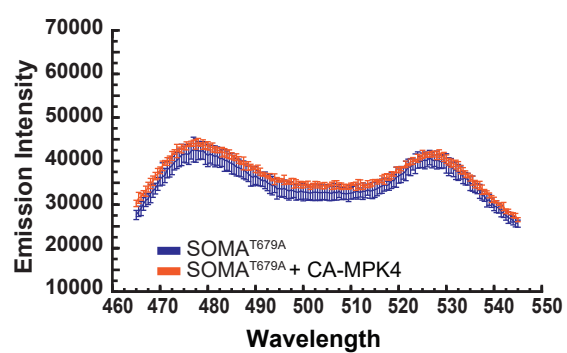**Figure S1**

Supplement: Supplementary file 1 [file ijms-21-05350-s001.zip › Supp. Fig. 1.pdf]

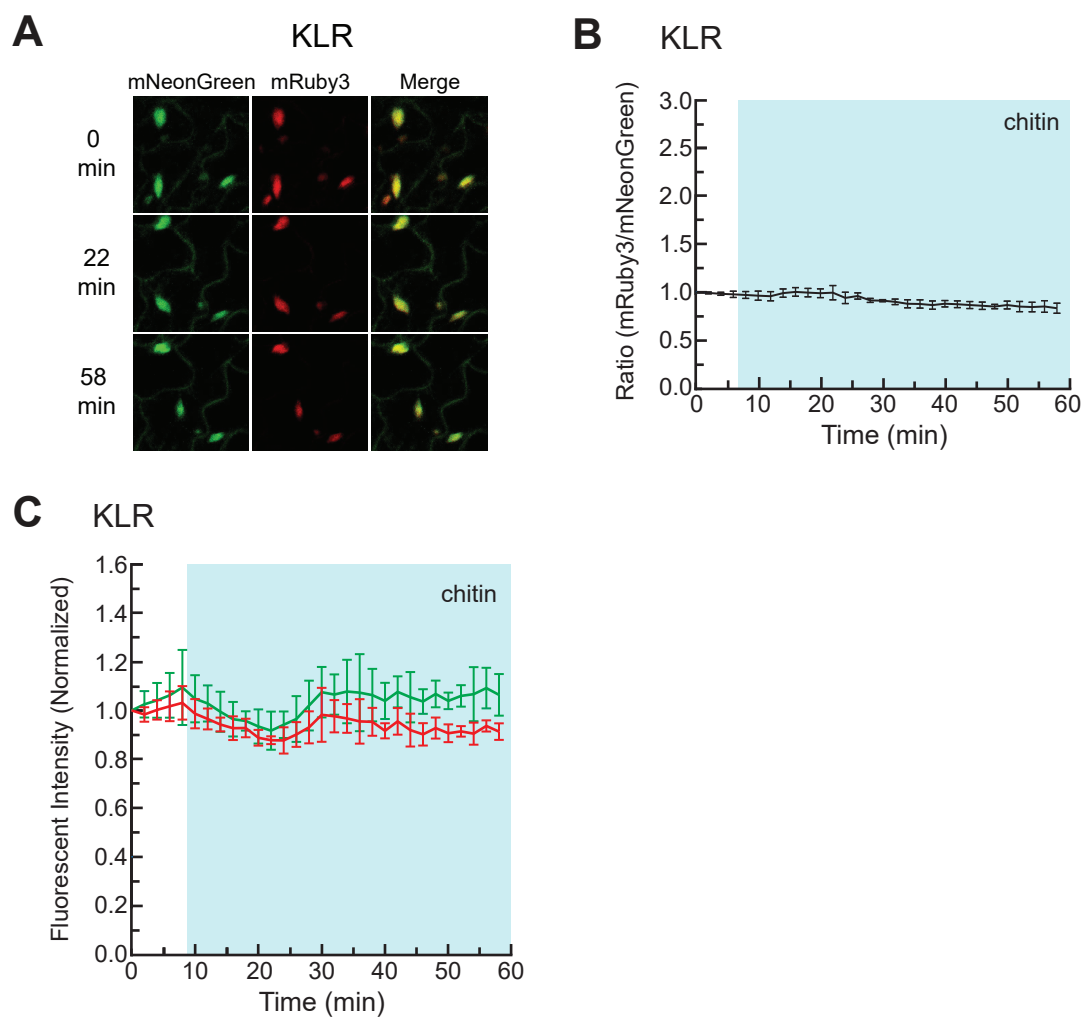

**Figure S2**

Supplement: Supplementary file 1 [file ijms-21-05350-s001.zip › Supp. Fig. 2.pdf]

**A** KLR-MKP1

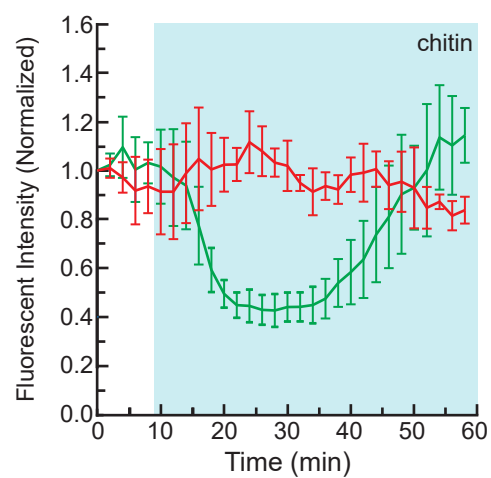

**B** KLR<sup>AA</sup>-MKP1

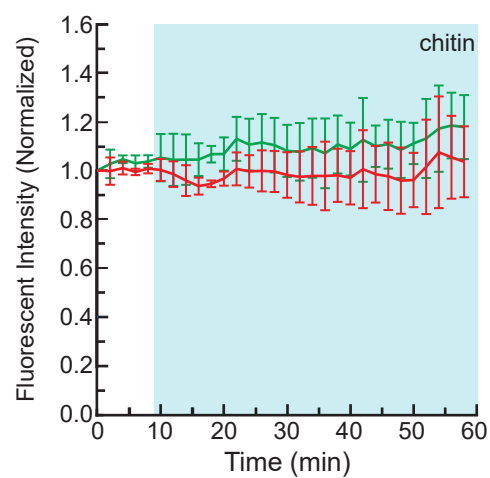

**C** KLR-MPK1

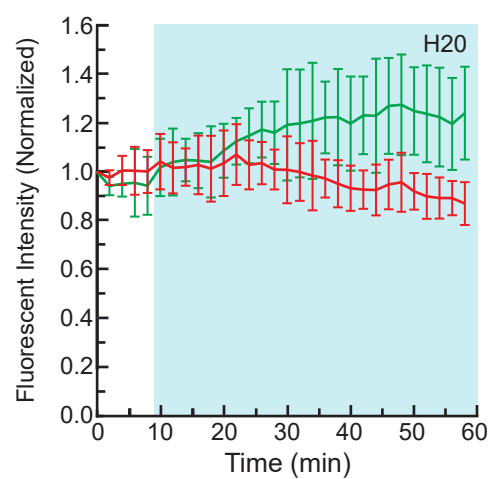

**D** KLR<sup>AA</sup>-MKP1

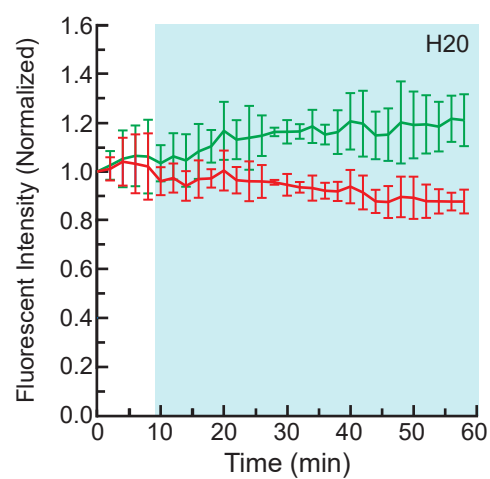

**Figure S3**

Supplement: Supplementary file 1 [file ijms-21-05350-s001.zip › Supp. Fig. 3.pdf]

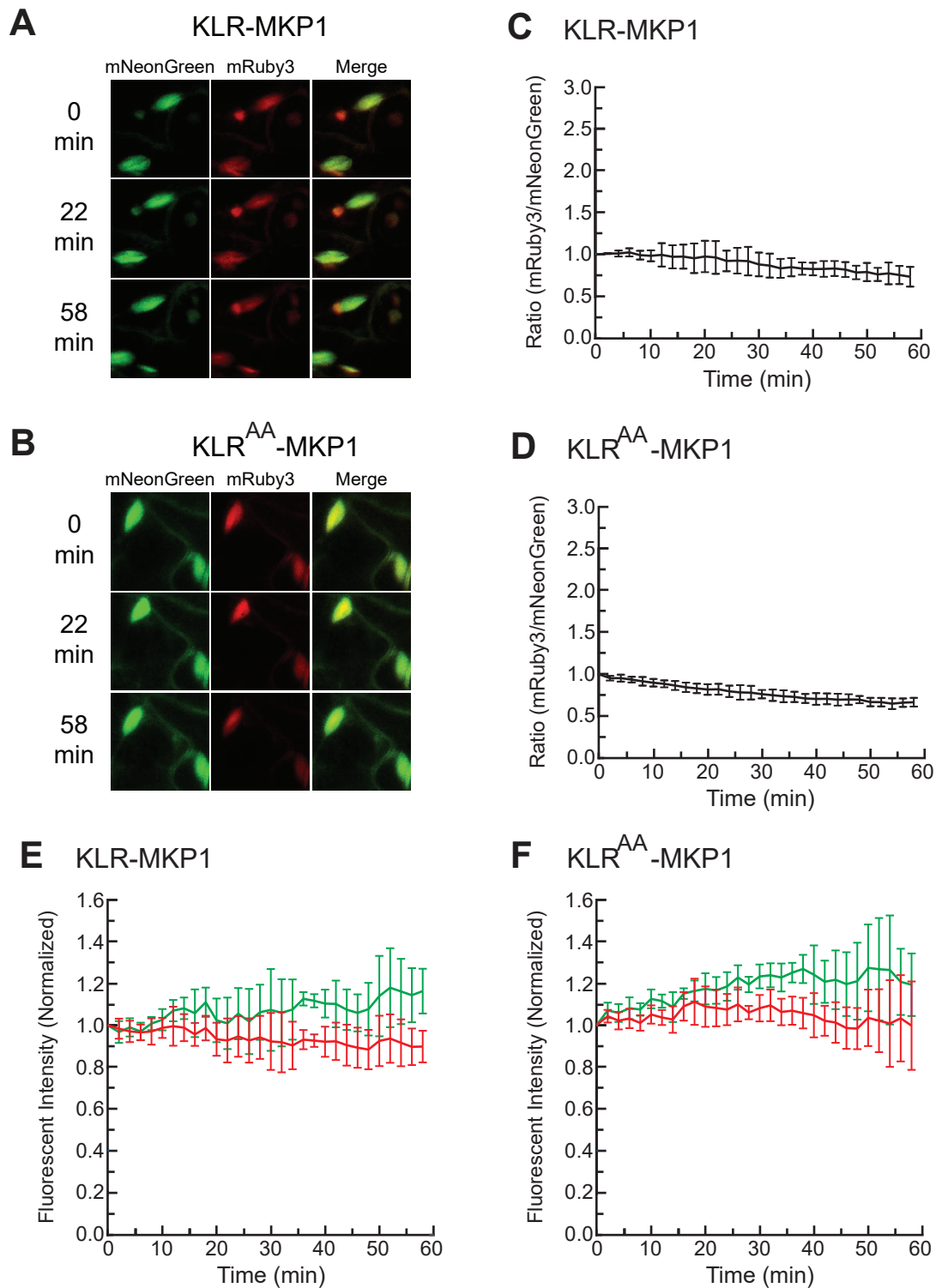

**Figure S4**

Supplement: Supplementary file 1 [file ijms-21-05350-s001.zip › Supp. Fig. 4.pdf]

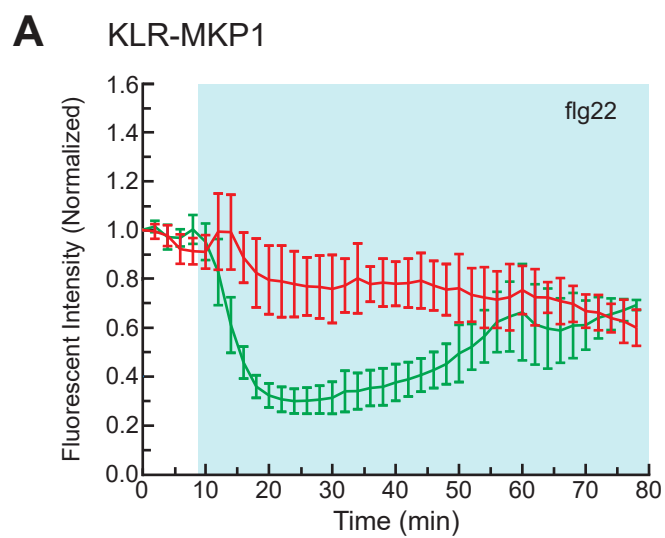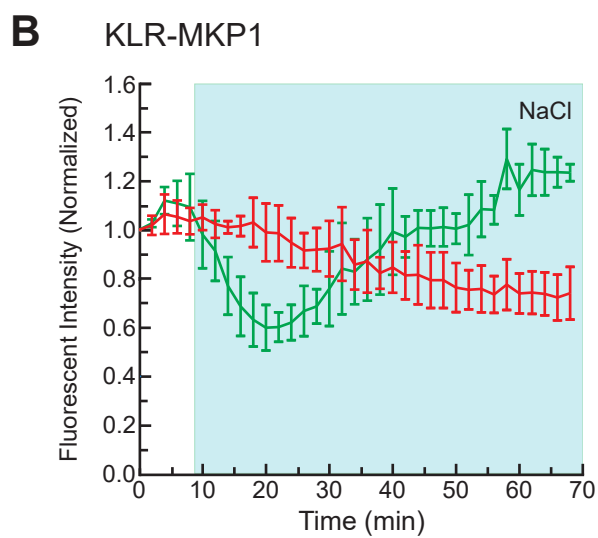

**Figure S5**

Supplement: Supplementary file 1 [file ijms-21-05350-s001.zip › Supp. Fig. 5.pdf]

**A** KLR-AP2C1

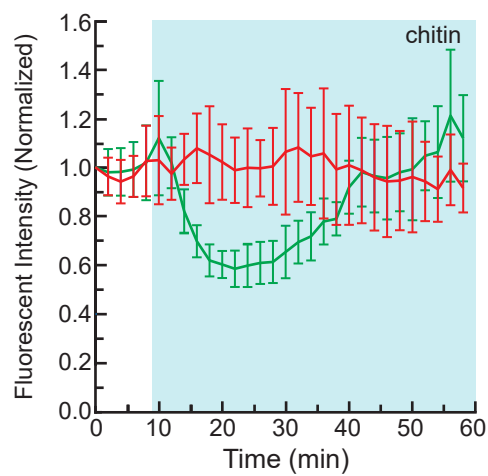

**B** KLR<sup>AA</sup>-AP2C1

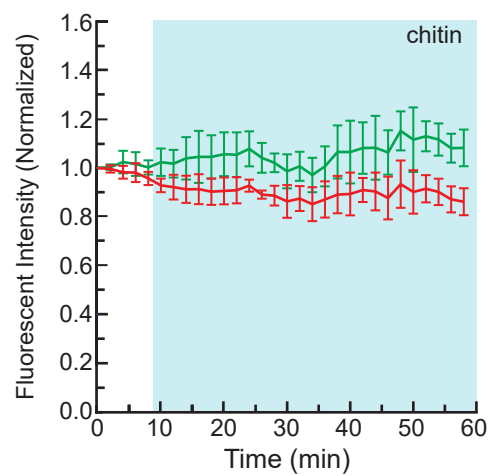

**C** KLR-AP2C1

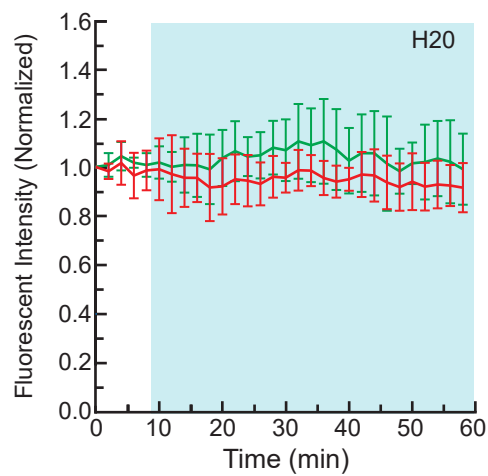

**D** KLR<sup>AA</sup>-AP2C1

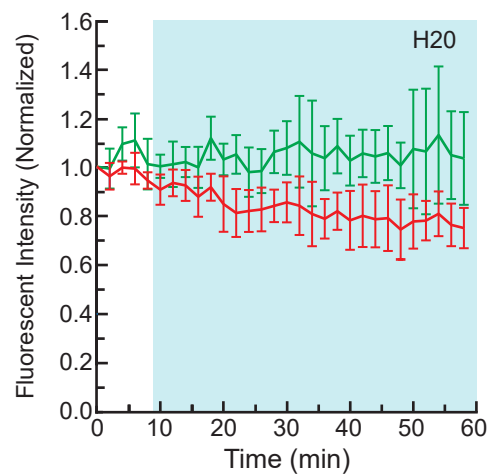

**Figure S6**

Supplement: Supplementary file 1 [file ijms-21-05350-s001.zip › Supp. Fig. 6.pdf]

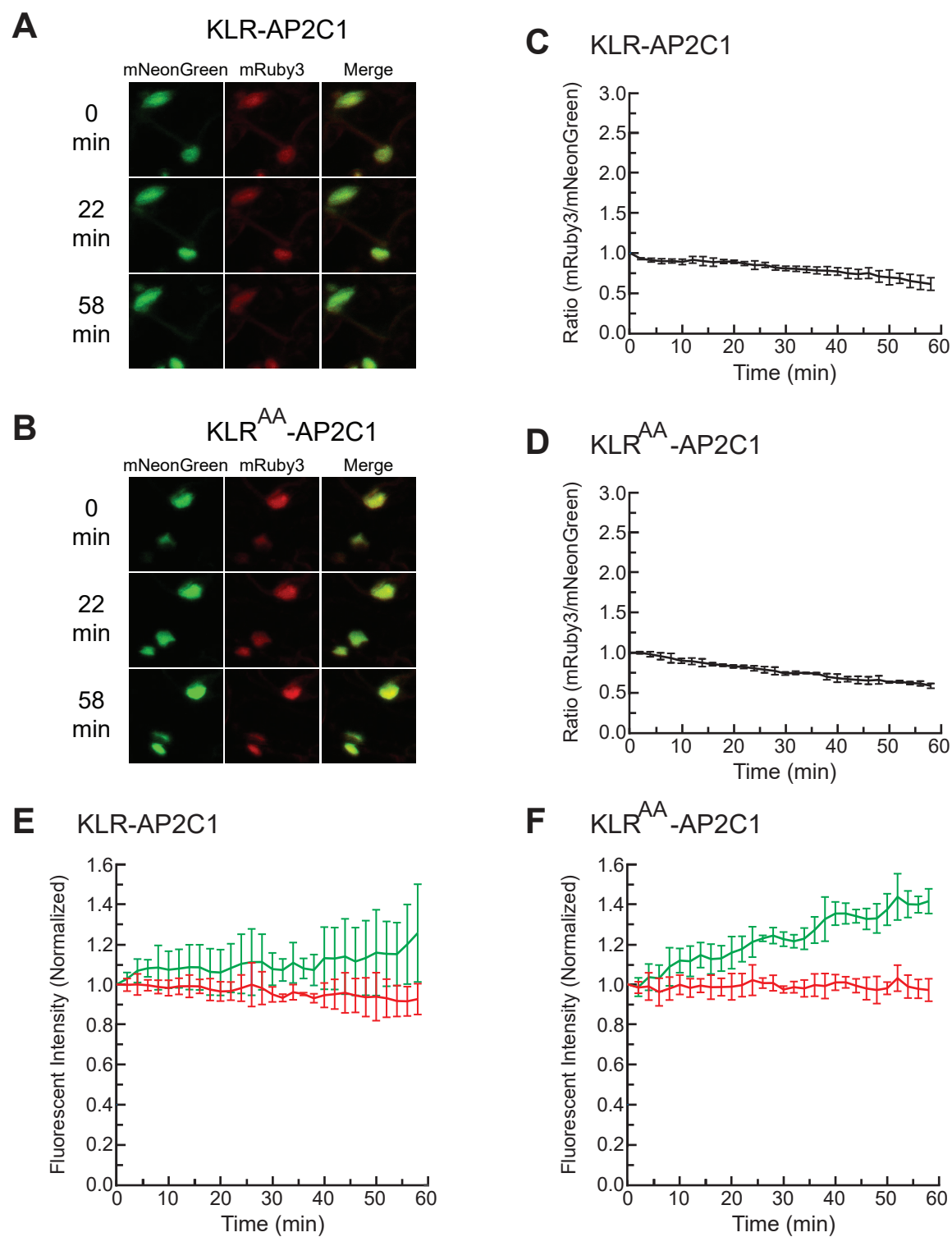

**Figure S7**

Supplement: Supplementary file 1 [file ijms-21-05350-s001.zip › Supp. Fig. 7.pdf]
